# Preparation and Antitumoral Activity of Au-Based Inorganic-Organometallic Nanocomposites

**DOI:** 10.3389/fchem.2019.00060

**Published:** 2019-02-08

**Authors:** Mariona Dalmases, Andrea Pinto, Petra Lippmann, Ingo Ott, Laura Rodríguez, Albert Figuerola

**Affiliations:** ^1^Departament de Química Inorgànica i Orgànica, Secció de Química Inorgànica, Universitat de Barcelona, Barcelona, Spain; ^2^Institut de Nanociència i Nanotecnologia (IN2UB), Universitat de Barcelona, Barcelona, Spain; ^3^Institute of Medicinal and Pharmaceutical Chemistry, Technische Universität Braunschweig, Braunschweig, Germany

**Keywords:** nanoparticles, gold, biological activity, organometallic, hybrid

## Abstract

The synergy between gelator molecules and nanostructured materials is currently a novel matter of study. The possibility to carefully design the skeleton of the molecular entity as well as the nanostructure's morphological and chemical features offers the possibility to prepare a huge variety of nanocomposites with properties potentially different than just the sum of those of the individual building blocks. Here we describe the synthesis and characterization of nanocomposites made by the unconventional combination of phosphine-Au(I)-alkynyl-based organometallic gelating molecules and plasmonic Au nanoparticles. Our results indicate that the interaction between the two moieties leads to a significant degree of aggregation in both hydrophilic and hydrophobic media, either when using DAPTA or PTA-based organometallic molecules, with the formation of a sponge-like hybrid powder upon solvent evaporation. The biological activity of the nanocomposites was assessed, suggesting the existence of a synergetic effect evidenced by the higher cytotoxicity of the hybrid systems with respect to that of any of their isolated counterparts. These results represent a preliminary proof-of-concept for the exploitation of these novel nanocomposites in the biomedical field.

## Introduction

Metal-organic molecules have attracted much interest in the last decade due to their ability to spontaneously form supramolecular assemblies and metal-organic gels through the establishment of weak non-covalent intermolecular bonds of different nature (Zhang and Su, 2013). In particular, highly luminescent Au(I)-alkynyl-based organometallic complexes have revealed as excellent building blocks for this purpose, forming fibers, gels and other sorts of shape-controlled supramolecular structures, depending on their specific chemical formula and on the reaction conditions, with enhanced optical properties (Lima and Rodríguez, 2015). Noteworthy, Au(I)-alkynyl-based complexes can form aurophilic intra and intermolecular bonds between Au(I) ions, which do not only reinforce the stability of the assembly, but they also play a crucial role in defining the luminescent properties of the final product (Rodríguez et al., 2012; Schmidbaur and Schier, 2012)

On the other hand, phosphine-Au(I) compounds have often revealed themselves as cytotoxic species with interesting biological activity against tumor cell growth (Lima and Rodriguez, 2011). Although most of the studied phosphine-Au(I) metallodrugs contain a thiolate group directly attached to the Au(I) ion, phosphine-Au(I)-alkynyl complexes have been also observed to perform as antitumoral agents in a few cases (Chui et al., 2009; Schuh et al., 2009; Vergara et al., 2010; Meyer et al., 2012). Our group has recently reported some promising results on this topic (Meyer et al., 2013; Arcau et al., 2014; Andermark et al., 2016; Gavara et al., 2016).

In the field of nanoparticles or nanochemistry, Au(I)-alkynyl-based organometallic complexes have been successfully employed as molecular precursors for the synthesis of uniform Au nanoparticles, by means of their controlled thermal decomposition at relatively low temperatures (Aguiló et al., 2013; Ballesteros et al., 2014; Muhich et al., 2015). These studies were motivated by the possibility to obtain size and shape homogeneous metallic nanostructures under soft reaction conditions with interesting plasmonic properties. Nevertheless, recent studies are focusing more into the possible synergy established between gelator molecules and different types of nanostructured materials, i.e., inorganic nanoparticles, carbon nanotubes, etc (Das et al., 2012). In this regard, our group has recently reported on the synergy established between phosphine-Au(I)-alkynyl-based complexes and metallic nanoparticles, which allowed for the complete solubilization of hydrophobic colloids into water as well as for their plasmon tuning (Dalmases et al., 2016). Considering the enormous amount of highly designed systems potentially achieved, the preparation of hybrid organometallic nanocomposites should be strongly pursued, since they might open the doors for the use of a new set of engineered materials with multiple and enhanced properties useful in different fields.

Au nanoparticles have experienced a huge growing interest in the last decades due to their fascinating optical properties that are unfolded in the shape of an intense absorption band in the visible or near infrared region (NIR), known as the Localized Surface Plasmon Resonance (LSPR) (Motl et al., 2014). The origin of this phenomenon in metal nanostructures like Au or Ag is well-understood and is grounded on the collective excitation of surface electrons by means of the electric field of incident light, resulting in a coherent surface-confined oscillation with a resonant frequency that strongly depends on the material itself, as well as on the size, shape, dielectric environment, and separation distance of nanoparticles (NPs). The high level of control achieved in their synthesis and morphological features, as well as on their optical properties, have made of Au nanoparticles an interesting material particularly involved in both the photodiagnostics and photothermal therapy of cancers and other main diseases (Boisselier and Astruc, 2009).

Based on all this, the combination of phosphine-Au(I)-alkynyl-based organometallic complexes and plasmonic nanoparticles seems a promising approach for the design of new theranostic agents, able to perform simultaneously diagnostic and therapeutic tasks when required during the medical treatment. Nevertheless, there are no examples reported on the synthesis of such type of nanocomposites besides our previous work (Dalmases et al., 2016), and thus their potential biological activity is yet to unravel.

**Scheme 1:**
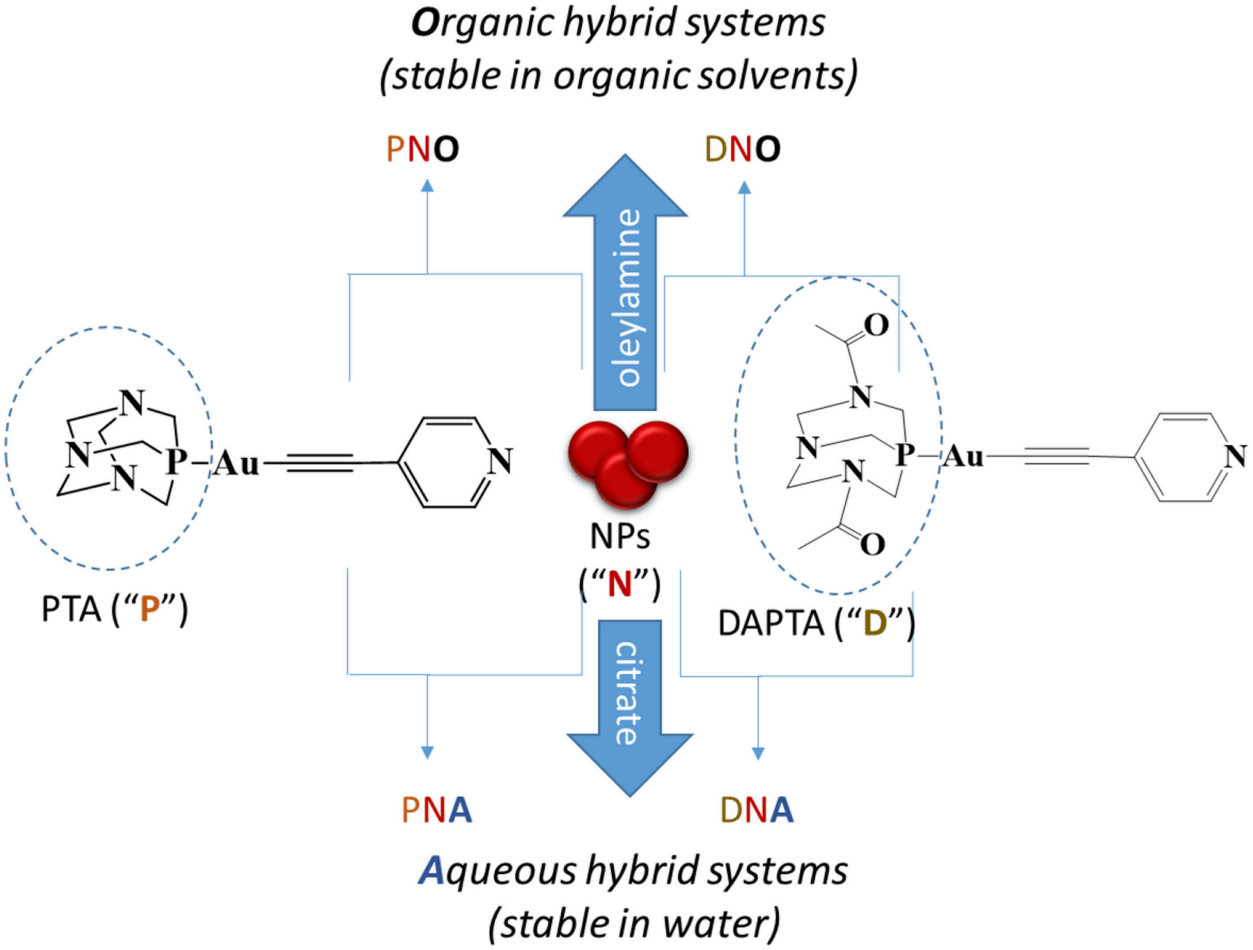
Types of nanocomposites prepared and description of the nomenclature used for each of them. That is, “N” indicates nanoparticles; “P,” PTA phosphine derivatives; “D,” DAPTA phosphine derivatives; “O,” organic medium and “A,” aqueous medium.

In this work, we report on the preparation of novel hybrid nanosystems combining Au nanoparticles and phosphine-Au(I)-alkynyl-based supramolecular structures with the aim of assessing their potential as efficient antitumoral agents, with special emphasis on the identification of synergetic effects between the metallic and the molecular moieties with respect to their isolated counterparts. The antitumoral activity depends on the hydro or lipophilicity of the drug and thus we decided to work with two different types of Au nanoparticles which differ basically on the hydrophilicity of their initial stabilizing molecule after synthesis, being either citrate anions or oleylamine molecules for the hydrophilic and the hydrophobic cases, respectively, as shown in Scheme 1. Our results show that significantly lower IC_50_ values are found for all the hybrid systems prepared with respect to their individual counterparts (NPs and organometallic structures), representing a good proof-of-concept for the future development and study of these new type of biologically active nanocomposites.

## Materials and Methods

### General Procedures

Commercial reagents 1,3,5-triaza-7-phosphatricyclo[3.3.1.13.7]decane (PTA; 97%, Aldrich), 3,7-diacetyl-1,3,7-triaza-5-phosphabicyclo[3.3.1]nonane (DAPTA; 97%, Aldrich), Gold(III) chloride trihydrate (HAuCl_4_·3H_2_O, ≥99.9, Aldrich), trisodium citrate dihydrate (HOC(COONa)(CH_2_COONa)_2_·2H_2_O, Aldrich), oleylamine (OLAm, 70%, Aldrich), oleic acid (OLAc, ≥99%, Aldrich), 1-octadecene (ODE, 90%, Aldrich), 2-Propanol (iPrOH, for HPLC, VWR Chemicals), and chloroform (CHCl_3_, 99.2%, VWR Chemicals) have been used as received. Literature methods have been used to prepare [Au(C≡ C–C_5_H_4_N)(PTA)] (Gavara et al., 2013). and [Au(C≡ C–C_5_H_4_N)(DAPTA)] (Aguiló et al., 2013) compounds, that from now on will be named PTA Au(I) complex and DAPTA Au(I) complex, respectively, for the sake of simplicity.

### Hydrophilic Au NPs

The synthesis of hydrophilic Au NPs was based on that reported by Turkevish and coworkers (Turkevich et al., 1953). Briefly, 8.8 mg HAuCl_4_·3H_2_O (0.02 mmol) were dissolved in 60 mL of deionized water and the solution was heated to the boiling point. Once the temperature was reached, 5.2 mL of an aqueous solution of sodium citrate at 2% was added and let the reaction for 20 min at this temperature. The color of the solution changed from yellow to dark red as a result of the formation of gold nanoparticles.

### Hydrophobic Au NPs

The synthesis of hydrophobic Au NPs was adapted from that described by Yu and co-workers as follows: (Yu et al., 2005) A mixture of ODE (30 mL), OLAc (4.5 mL), and OLAm (5.5 mL) was stirred in a 100 mL three-necked flask for 20 min at 120°C under vacuum. Meanwhile, in a glovebox HAuCl_4_·3H_2_O (120 mg, 0.30 mmol) was dissolved in ODE (7.5 mL) and OLAm (1.5 mL) in an auxiliary vial. Once the purge was complete and the system under N_2_, the solution of gold precursor was injected into the flask and temperature was fixed at 150°C. After 30 min of reaction, the heating was stopped, and the solution was washed with 2 equivalent volumes of iPrOH, and centrifuged at 4,500 rpm for 4 min. The product was redispersed in chloroform.

## Synthesis of Hybrid Organometallic Supported NPs

### Au-DAPTA Nanocomposites in Organic Media (DNO Systems)

The functionalization of hydrophobic Au nanoparticles with DAPTA was performed by a simple reaction at room temperature in chloroform. 4.2 mg of DAPTA (7.94 × 10^−3^ mmol) were dissolved in 8 mL chloroform and mixed with a solution of hydrophobic Au nanoparticles dispersed in chloroform. The solution was shaken for 24 h. After this time, the solution was centrifuged and the resultant precipitate was redispersed in chloroform.

### Au-PTA Nanocomposites in Organic Media (PNO Systems)

The functionalization of hydrophobic Au nanoparticles with PTA was adapted from the protocol described above but using 1.3 mg (2.85 × 10^−3^ mmol) of PTA instead of DAPTA.

### Au-DAPTA and Au-PTA Nanocomposites in Aqueous Media (DNA and PNA Systems, Respectively)

The synthesis of Au-DAPTA (or -PTA) hydrophilic nanocomposites were done following the same procedure described for the nanocomposites in organic media but substituting the chloroform for deionized water.

Three different samples of each nanocomposite were synthesized using different amounts of Au NPs. The volumes of Au NPs solution used in each case are compiled in Table 1.

**Table 1:** Volumes of Au NP stock solution used in each synthesis.

**Sample**	**Volume of Au NP stock solution**
DNO_1	540 μL hydrophobic Au NPs (CHCl_3_)
DNO_2	270 μL hydrophobic Au NPs (CHCl_3_)
DNO_3	54 μL hydrophobic Au NPs (CHCl_3_)
DNA_1	1600 μL hydrophilic Au NPs (H_2_O)
DNA_2	800 μL hydrophilic Au NPs (H_2_O)
DNA_3	160 μL hydrophilic Au NPs (H_2_O)
PNO_1	540 μL hydrophobic Au NPs (CHCl_3_)
PNO_2	270 μL hydrophobic Au NPs (CHCl_3_)
PNO_3	54 μL hydrophobic Au NPs (CHCl_3_)
PNA_1	1600 μL hydrophilic Au NPs (H_2_O)
PNA_2	800 μL hydrophilic Au NPs (H_2_O)
PNA_3	160 μL hydrophilic Au NPs (H_2_O)

*The concentration of Au NPs in aqueous stock solution was 0.015 μM and in organic stock solution 2.33 μM*.

## Physical Measurements

### Transmission Electron Microscopy (TEM)

Au NPs were prepared for observation by TEM by dilution in chloroform/water followed by sonication. A droplet of the solution was then poured in holey carbon covered copper TEM grids. A JEOL 2000 FX II conventional TEM operating at an accelerating voltage of 80 kV was used.

### SEM

Scanning electron microscopy (SEM) was carried out at 20 kV using J-7100F (Jeol) equipped with a thermal field electron source.

### DLS

Dynamic Light Scattering (DLS) measurements were carried out in a Zetasizer NanoS Spectrometer. The samples were measured in quartz cuvettes.

### Infrared Spectroscopy

IR spectra were recorded with a FTIR 520 Nicolet Spectrophotometer. For the measurements, a pellet of a mixture of the sample and KBr was used.

### UV-Vis Absorbance Spectroscopy

A Cary 100 Scan 388 Varian UV/Vis spectrophotometer was used with quartz cuvettes for optical characterization.

### ICP-AES

The compositions and concentrations of the nanoparticles solutions were determined by inductively coupled plasma atomic emission spectroscopy (ICP-AES). The measurements were performed with an Optima 3200 RL PerkinElmer spectrometer. For the measurements, 50 mL of the solutions was precipitated in MeOH and redispersed in CHCl_3_. The solution was evaporated in an oven overnight at 90°C. Aqua regia (2.5 mL) and H_2_O_2_ (0.7 mL) were added to the precipitate before the vial was sealed and then heated to 90°C for 72 h. The resulting solution was transferred to a 25 mL volumetric flask and diluted with Milli-Q water.

### NMR

^1^H NMR [δ(TMS) = 0.0 ppm], ^31^P{^1^H} NMR [δ(85% H_3_PO_4_) = 0.0 ppm] spectra have been obtained on a Varian Mercury 400 and Bruker 400.

### Small-Angle X-Ray Scattering (SAXS)

SAXS was performed on the NCD-SWEET beamline at the ALBA Synchrotron at 12.4 keV, and the distance sample/detector was 6.2 m to cover the range of momentum transfer of 0.028 < *q* <2.56 nm^−1^. The data were collected on a Pilatus3S 1M detector with a pixel size of 172.0 × 172.0 μm^2^. The exposure time was 30 s. The q-axis calibration was obtained by measuring silver behenate (Huang et al., 1993). The program pyFAI was used to integrate the 2D SAXS data into 1D data (Kieffer and Karkoulis, 2013). The data were then subtracted by the background using PRIMUS software (Konarev et al., 2003). The maximum particle dimension Dmax and the pair distance distribution function P(r) were determined with GNOM (Svergun, 1992). The low-resolution structure of the aggregates was reconstructed *ab initio* from the initial portions of the scattering patterns using the program DAMM (Svergun, 1999; Krebs et al., 2004).

### Cell Culture and Antiproliferative Effects

MDA-MB-231 breast adenocarcinoma and HT-29 colon carcinoma were maintained in DMEM high glucose (PAA) supplemented with 50 mg/L gentamycin and 10% (V/V) fetal calf serum (FCS) at 37°C under 5% CO_2_ atmosphere and passaged every 7 days. Antiproliferative effects were determined as follows: a volume of 100 μL of a 38,000 cells/ml (HT-29) or 40000 cells/ml (MDA-MB-231) suspension were seeded into 96-well plates and incubated for 48 or 72 h at 37°C/5% CO_2_. After the incubation period the cells of one individual plate were fixed by addition of 100 μL of a 10% glutaraldehyde solution per well. After 30 min. One hundred and Eighty Microliter PBS were added and the plate was stored at 4°C (t_0_ plate) until further procedures (see below). Nanoparticle suspensions were diluted 1:100 with cell culture medium to the final test concentrations. In the remaining plates the medium was replaced by the medium containing the nanoparticles or solvent control (water or CHCl_3_). Then the plates were incubated for 72 h (HT-29) or 96 h (MDA-MB-231) at 5% CO_2_/37°C. The medium was removed and the cells were treated with 100 μl of a 10% glutaraldehyde solution. Afterwards the cells of all plates (including the t_0_ plate) were washed with 180 μL PBS and stained with 100 μL of a 0.02% crystal violet solution for 30 min. The crystal violet solution was removed and the plates were washed with water and dried. A volume of 180 μL of ethanol 70% was added to each well and after 2–3 h of gentle shaking the absorbance was measured at 595 nm in a microplate reader (Victor X4, PerkinElmer). The mean absorbance value of the t_0_ plate was subtracted from the absorbance values of all other absorbance values in order to correct for the initial cell biomass. The IC_50_-values were calculated as the concentrations reducing the cellular proliferation in comparison with the solvent control by 50%.

## Results and Discussion

### Synthesis and Characterization

**Figure 1:**
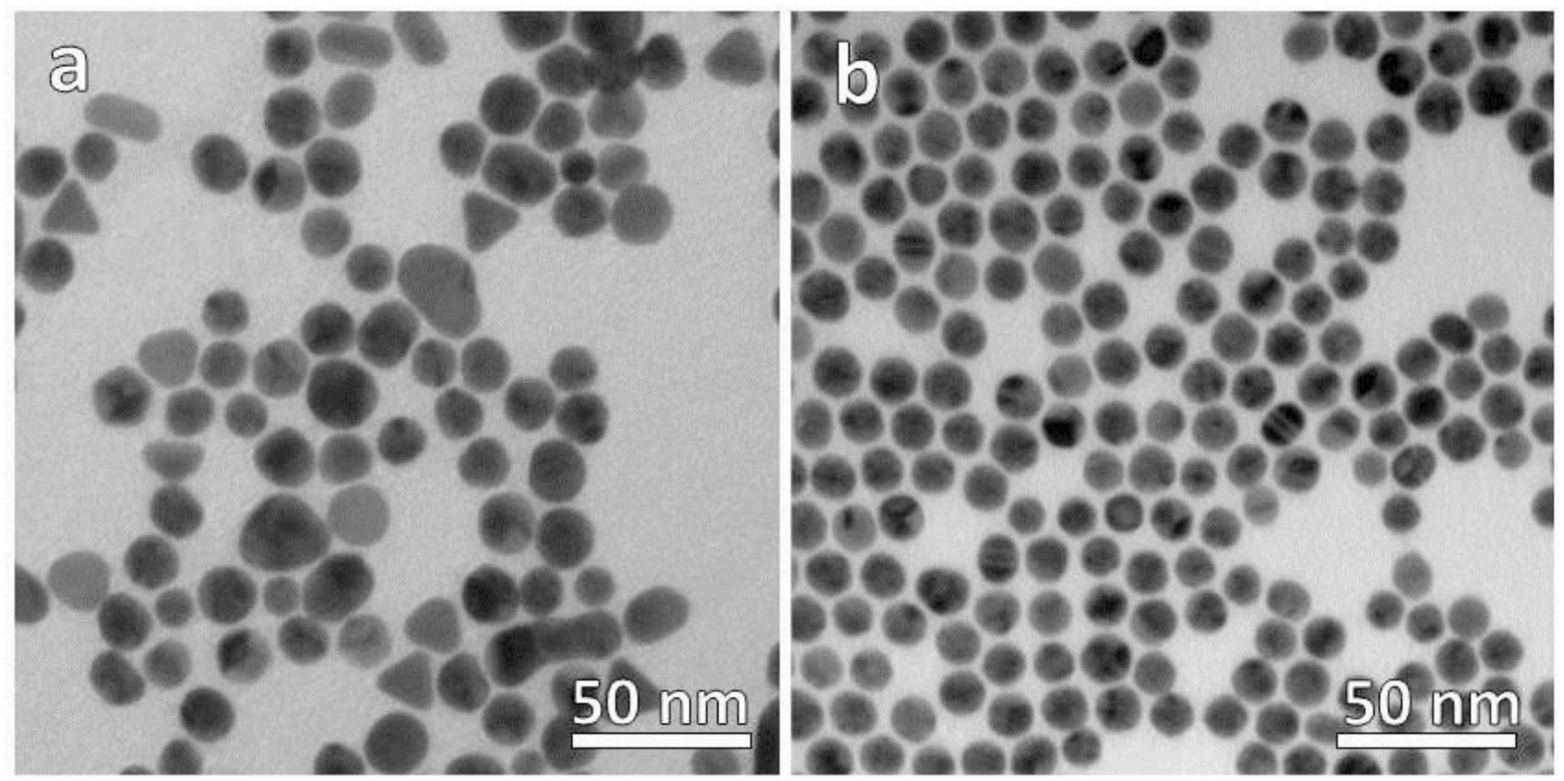
TEM micrographs of **(a)** hydrophilic citrate-capped and **(b)** hydrophobic oleylamine-capped Au NPs.

Our synthetic strategy started by preparing two samples of Au NPs capped by hydrophilic and hydrophobic stabilizing ligands separately, i.e., citrate anions and oleylamine molecules, respectively (see Materials and Methods section). TEM micrographs of the as-prepared samples allowed to evaluate the morphological and size distribution of the NPs in the two samples. The hydrophilic sample consists of a collection of nanoparticles with a significant distribution of shapes including spherical (the most abundant), triangular, and elongated nanoparticles of low aspect ratio as shown in Figure 1a. On the contrary, Figure 1b is a TEM representative image of the hydrophobic sample in which only spherical faceted nanoparticles are observed. The size distribution was also much narrower in the hydrophobic sample, even though both samples showed an average diameter of ca. 14 nm and their deposition on the TEM grid suggests that the colloids are well-dispersed in solution without evidences of aggregation.

**Figure 2:**
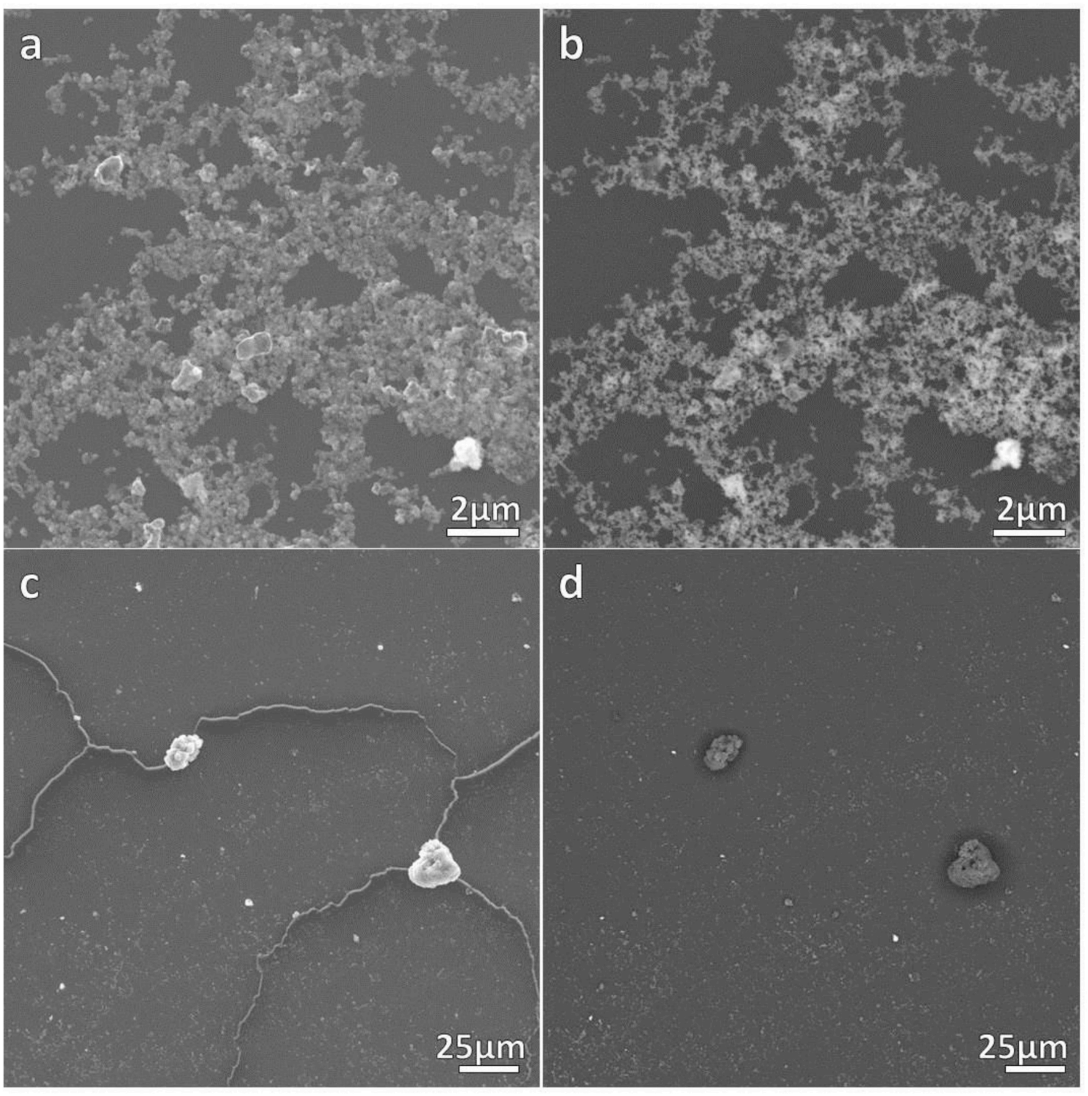
SEM micrographs of DNO_1 sample. **(a,c)** contain topographical micrographs recorded with secondary electrons, while **(b,d)** contain Z-sensitive micrographs recorded with backscattered electrons.

**Figure 3:**
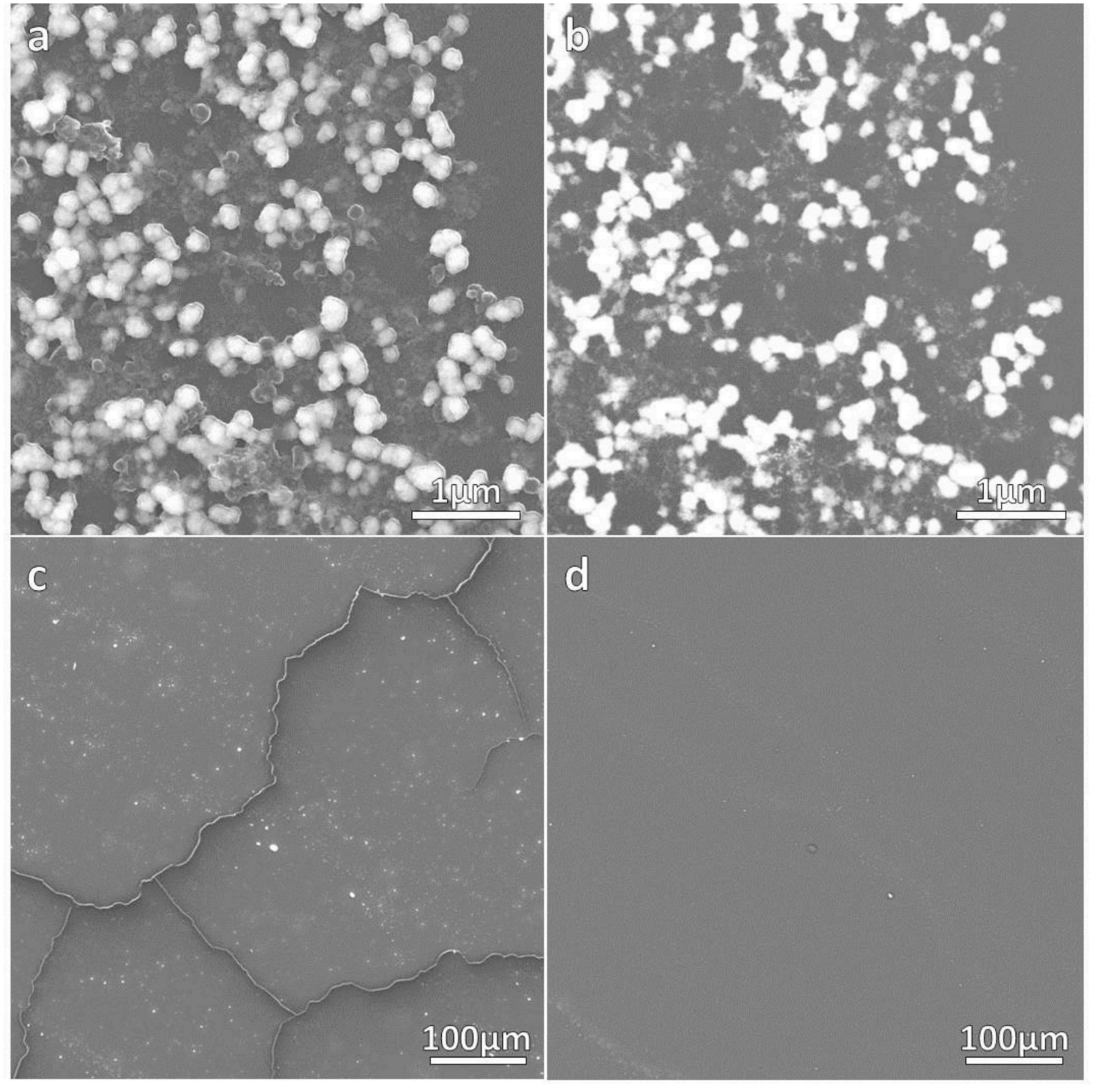
SEM micrographs of PNA_1 sample. **(a,c)** contain topographical micrographs recorded with secondary electrons, while **(b,d)** contain Z-sensitive micrographs recorded with backscattered electrons.

The correct formation of the resulting nanocomposites was evidenced by SEM micrographs as depicted in Figures 2, 3 corresponding to DNO_1 and PNA_1 samples, made of DAPTA Au(I) complex in chloroform and PTA Au(I) complex in water respectively, and both with the highest amount of Au NPs in the reaction medium. The images suggest that the samples are mainly constituted by aggregates of Au NPs, with sizes ranging between 100 and 300 nm approximately that are well-interconnected between them forming a kind of sponge-like 3D network. The individual aggregates are likely surrounded by a shell of the organometallic complex. Indeed, a quantitative shrinkage of the aggregates is evident when comparing topographic images in panels (a) and (c) in the figures, recorded with secondary electrons, with the corresponding Z-sensitive images in panels (b) and (d), recorded with backscattered electrons. The significant reduction in volume of the clusters between the two related images is indicative of the presence of an organic shell around the metallic NPs. Besides the presence of the organometallic-NP composites, few long fibers are also observed at low magnifications (Figures 2c,d, 3c,d). The vanishing of these fibers in images recorded using backscattered electrons confirms their organic origin, suggesting that they are formed as a result of the supramolecular interactions between free molecules of the organometallic moiety, as it was observed in previous studies of these organometallic complexes (Aguiló et al., 2013), and that they do not incorporate Au NPs in their structure. Generally speaking, the observed trend with amount of Au NPs was the same, independently of the organometallic molecule and solvent used for the synthesis of the nanocomposites: the larger the amount of Au NPs used for the synthesis, the higher the number of composite aggregates encountered, while few or none nanocomposites were found in samples containing the lowest amount of Au NPs (samples XXX_3 of each group), as shown in Figures S1–S4 in the SI. Some large aggregates of approximately 20 μm can also be appreciated by SEM, although in a much lower frequency compared with the previous smaller ones. DLS measurements performed on these samples mainly confirm the average size of the aggregates, this being of a few hundreds of nanometers as observed by SEM, as well as the presence of a small population of microscale aggregates as indicated by SEM too (see Figure S5 in the SI). Noteworthy, DLS data evidence the presence of smaller aggregates with sizes ranging from 20 to 60 nm approximately depending on the sample, which cannot be discerned from the larger ones by previous SEM characterization.

**Figure 4:**
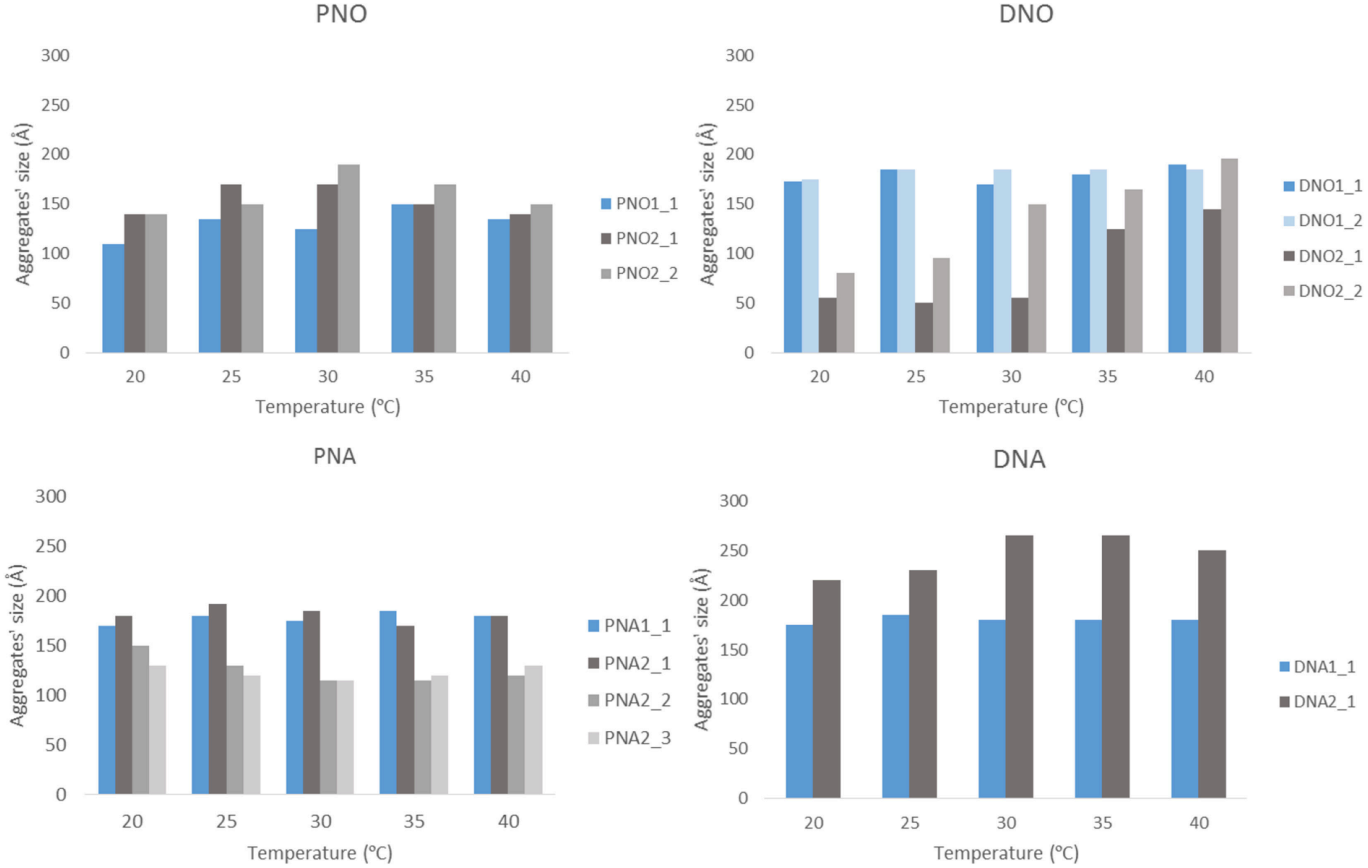
Representation of aggregates' size vs. temperature. The results are plotted separately depending on the phosphine and the medium. Blue bars stand for XXX_1 and gray bars for XXX_2 samples.

The size and shape of the aggregates were measured by SAXS for those samples prepared with the highest (XXX_1) and medium (XXX_2) amount of Au NP. Samples with less amount of Au NPs were not measured since previous SEM studies revealed the presence of no composites in the solutions. Each of the eight considered samples (DNO_1/2; DNA_1/2; PNO_1/2 and PNA_1/2) was measured as initially prepared and also with two additional diluted concentrations (i.e., with DNO_1_1 being the as-prepared sample, and DNO_1_2 and DNO_1_3 the samples derived from 1/500 and 1/1000 dilution), at different temperatures (from 20 to 40°C in a 5°C gradient, for better analysis of the aggregates' formation) and 1 week after their preparation to favor aggregation. The low-resolution structures were reconstructed *ab initio* from the scattering patterns using the DAMMIN program (see Materials and Methods section). The resulting size and morphologies profile are depicted in Figures 4 and Figures S6–S18 for all compounds that display significant aggregation. No significant aggregation was detected for the rest of the cases. Generally speaking, aggregates of sizes between 10 and 30 nm are measured by SAXS depending on the sample, which hardly agrees with the size of the aggregates observed and measured by analysis of SEM micrographs in Figures 2, 3, but on the opposite their sizes roughly coincide with the values observed for the smallest aggregates detected by DLS measurements and shown in Figure S5. The values obtained by DLS and SAXS point out the possibility that the relatively large aggregates (100–300 nm) observed by SEM might be formed by smaller subunits (10–60 nm), linked by organometallic domains, that are only appreciable by light and X-ray scattering experiments. From our experimental data it can be concluded that Au NPs are mainly wrapped within a layer of organometallic complex forming small metallic clusters of a few nanoparticles. Additionally, larger 3D aggregates based on non-covalent interactions are formed, which are responsible for the sponge-like structure observed. In general, it can be seen that temperature does not affect substantially the size of the aggregates. Only in the case of DNO2, a significant increase on the size is clearly detected in both diluted samples, DNO2_1 and DNO2_2. This can be ascribed to the higher contribution of hydrophobic interactions at higher temperatures, being DAPTA moieties (more soluble in water) avoiding the contact with organic solvent. Within each set of samples measured at different concentrations, the collected data indicates that no aggregation occurs for the most diluted samples (XXX_X_3), while appreciable degrees of aggregation were measured, in general, for the other two dilutions. This data could indicate the existence of a critical concentration of aggregates below which they lose their stability. Some other trends can be observed looking at this data regarding now the amount of Au NPs used in the reaction. That is, in organic medium, a light increase of size was detected for PNO_2_X samples containing a smaller amount of Au NPs or aggregates compared to analogous PNO_1_X samples. On the contrary, DNO_1_X samples give rise to the formation of larger structures compared to the DNO_2_X analogs. These observations could be rationalized based on the lower solubility of DAPTA (in DNO complexes) in organic medium with respect to PTA (in PNO derivatives). Following this reason, it makes sense to understand the contrary effect in water: smaller aggregates for samples PNA_2_X containing a smaller amount of Au NPs or aggregates, and larger aggregates for samples DNA_2_X containing also a smaller amount of Au NPs or aggregates were observed, since the higher solubility in water of DAPTA Au(I) complex makes the formation of aggregates more difficult in this solvent.

The infrared spectra of the nanocomposites display the expected bands both from the organometallic complex and from the stabilizing ligands of the NPs. The resulting broadening of the alkynyl moieties are indicative of Au···π interaction between the NPs' surface and the complex.

**Figure 5:**
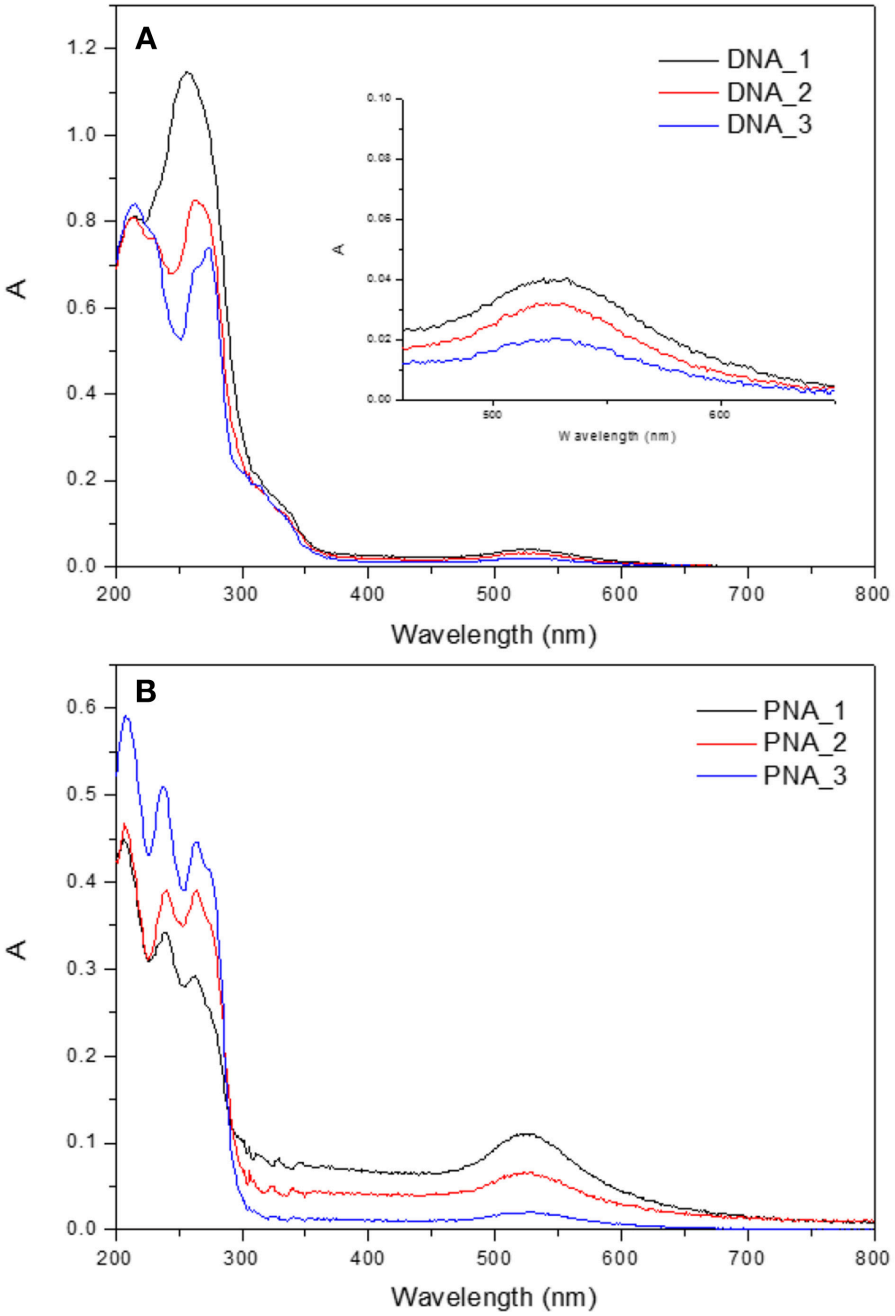
UV-Visible absorption spectra of **(A)** DNA_1-3 and **(B)** PNA_1-3 samples.

The UV/vis absorption spectra of the hybrid system present two different signals, as shown in Figure 5 for the hydrophilic nanocomposites with DAPTA and PTA. On the one hand, there is the band around 260 nm attributed to the Au(I) complex. On the other hand, the signal at 500–600 nm is assigned to the characteristic LSPR band of Au NPs. To better study the effect of the quantity of gold NPs added to the formation of hybrid system, absorption spectra of three different systems per organometallic molecule were recorded (DNA_1-3 and PNA_1-3). There is a change in the profile of the more energetically part of the absorption spectra, when the amount of nanoparticles used in the synthesis of the hybrid system increases, there is a loss of the vibrational shape in the band as an evidence of interaction between NPs and the corresponding organometallic complex (Ferrer et al., 2008).

### Biological Activity

The biological evaluation was focused on determining the effects against tumor cell growth in HT-29 colon carcinoma and MDA-MB-231 breast cancer (Table 2). Inspection of Table 2 let us retrieve some important conclusions: (i) Hybrid organic systems present higher cytotoxicity than the corresponding aqueous systems; (ii) hybrid systems present higher cytotoxicity than their respective counterparts, both organometallic complexes and NPs, being a nice cooperative effect than makes promising this encapsulation process regarding the study of their biological activity. However, the toxicity of the drug free organic NPs and the role of CHCl_3_ as solvent for redispersion has to be taken into account in future studies.

**Table 2:** IC_50_ values of the different hybrid systems and corresponding counterparts.

**Compound**	**HT-29 (μM)**	**MDA-MB-231 (μM)**
DNA1	6.14 ± 0.29	>10
DNA2	>6.4	>7.7
PNA1	>10	>10
PNA2	>10	>10
DNO1	3.41 ± 0.29	1.73 ± 0.44
DNO2	2.76 ± 0.23	>1.4
PNO1	1.82 ± 0.32	1.00 ± 0.17
PNO2	4.16 ± 0.38	2.02 ± 0.40
Org NPs	5.39 ± 0.89	6.01 ± 0.85
Aqueous NPs	>10	>10
AupyPTA^a^	56.09 ± 3.05	52.32 ± 4.76
AypyDAPTA^a^	74.78 ± 9.34	34.07 ± 3.51

a *From reference Arcau et al. (2014); mean values and standard errors of 2–3 independent experiments are presented*.

Experiments with non-tumor cells were not performed as tumor selectivity in the used assay could not be expected. By theory, nanoparticles would be enriched in tumor tissue by the EPR effect. This effect is not present in static tissue culture models, and thus tissue selectivity of nanoparticles can not be evaluated in this assay (Nakamura et al., 2016).

## Conclusions

The reaction of citrate and oleylamine-capped Au NPs with two different Au(I)-based organometallic complexes gives rise to the successful formation of nanocomposites soluble in either aqueous or organic media, forming large 3D sponge-like aggregates. SEM, DLS, SAXS, and spectroscopic techniques reveal useful for the verification of interaction between the corresponding building blocks. The lower IC_50_ values measured for nanocomposites compared to those of individual counterparts evidence their better biological activity against MDA-MB-231 and HT-29 tumor cells, opening a promising area of research in this field.

## Author Contributions

AF, LR, and IO designed the experiments. MD and AP carried out the synthesis and characterization of hybrid systems. PL carried out biological activity experiments. AF, LR, and IO supervised the project. All authors wrote and reviewed the manuscript.

### Conflict of Interest Statement

The authors declare that the research was conducted in the absence of any commercial or financial relationships that could be construed as a potential conflict of interest.
