# Supplementary material for: Preparation and Antitumoral Activity of Au-Based Inorganic-Organometallic Nanocomposites
Source: Front Chem. 2019 Feb 8;7:60. doi: 10.3389/fchem.2019.00060 (PMC6375849; doi:10.3389/fchem.2019.00060)
Supplement: Supplementary file 1 [file Data_Sheet_1.docx]

**Preparation and antitumoral activity of Au-based inorganic-organometallic nanocomposites**

Mariona Dalmases,^a,b,&^ Andrea Pinto,^a,b,&^ Petra Lippmann,^c^ Ingo Ott,^c^ Laura Rodríguez^a,b,*^ and Albert Figuerola.^a,b*^

^a^ Departament de Química Inorgànica i Orgànica, Secció de Química Inorgànica, Universitat de Barcelona, Martí i Franquès 1‐11, 08028 Barcelona, Spain. E-mail: [laura.rodriguez@qi.ub.es](mailto:laura.rodriguez@qi.ub.es); [albert.figuerola@qi.ub.es](mailto:albert.figuerola@qi.ub.es)

^b^ Institut de Nanociència i Nanotecnologia (IN2UB), Universitat de Barcelona, 08028 Barcelona, Spain

^c^ Institute of Medicinal and Pharmaceutical Chemistry, Technische Universität Braunschweig, Beethovenstrasse 55, 38106 Braunschweig, Germany.

Supporting Information


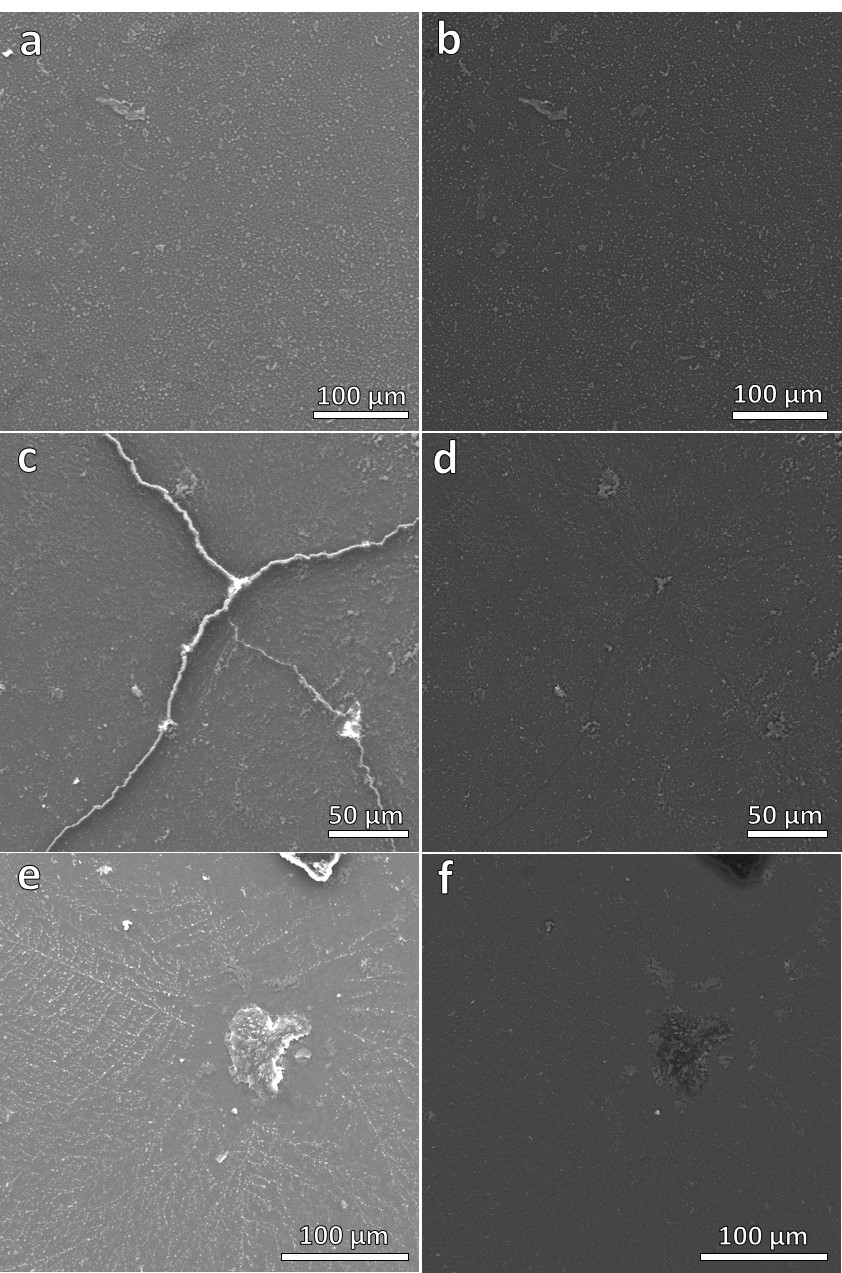


**Figure S1.** SEM micrographs for samples DNA_1 (a-b), DNA_2 (c-d) and DNA_3 (e-f). Topographical images in a, c and e panels were recorded with secondary electrons, while Z-sensitive images in panels b, d and f were recorded with backscattered electrons.


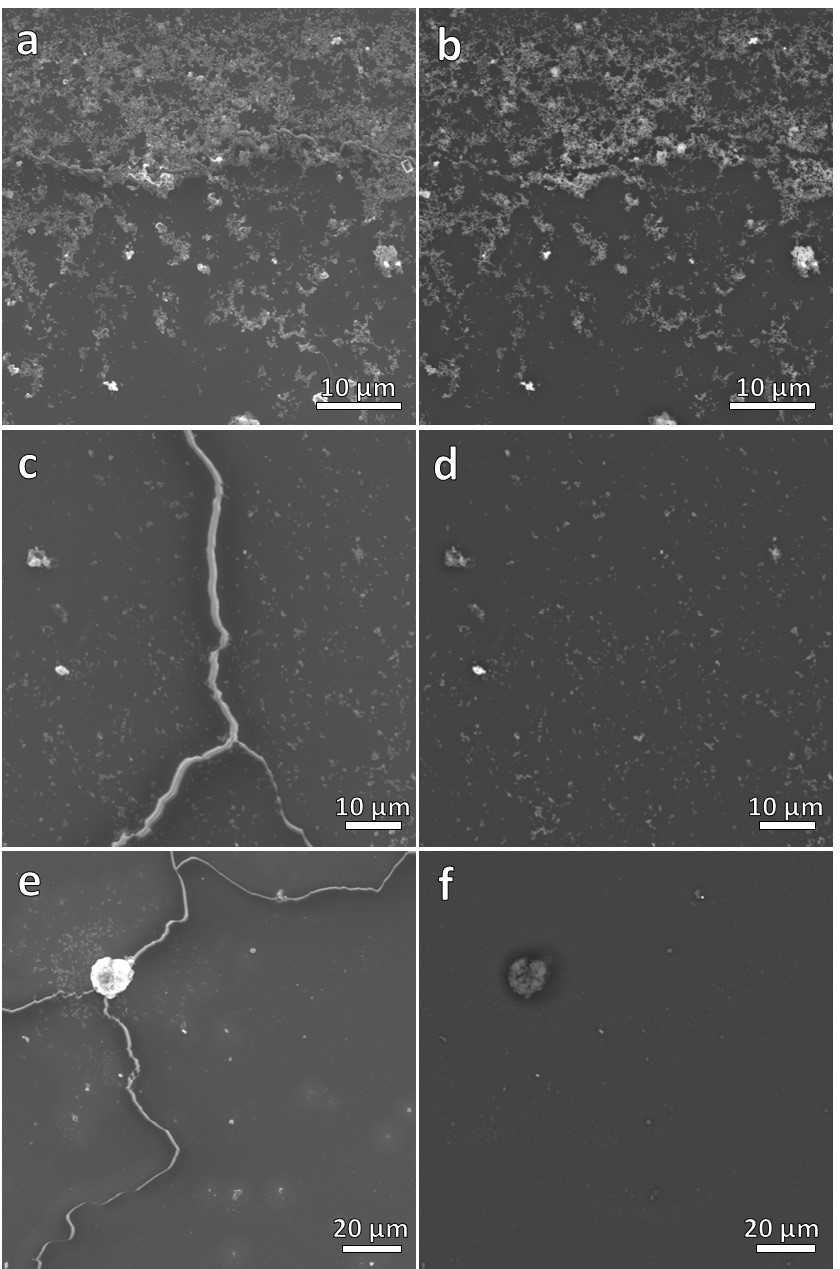


**Figure S2.** SEM micrographs for samples DNO_1 (a-b), DNO_2 (c-d) and DNO_3 (e-f). Topographical images in a, c and e panels were recorded with secondary electrons, while Z-sensitive images in panels b, d and f were recorded with backscattered electrons.


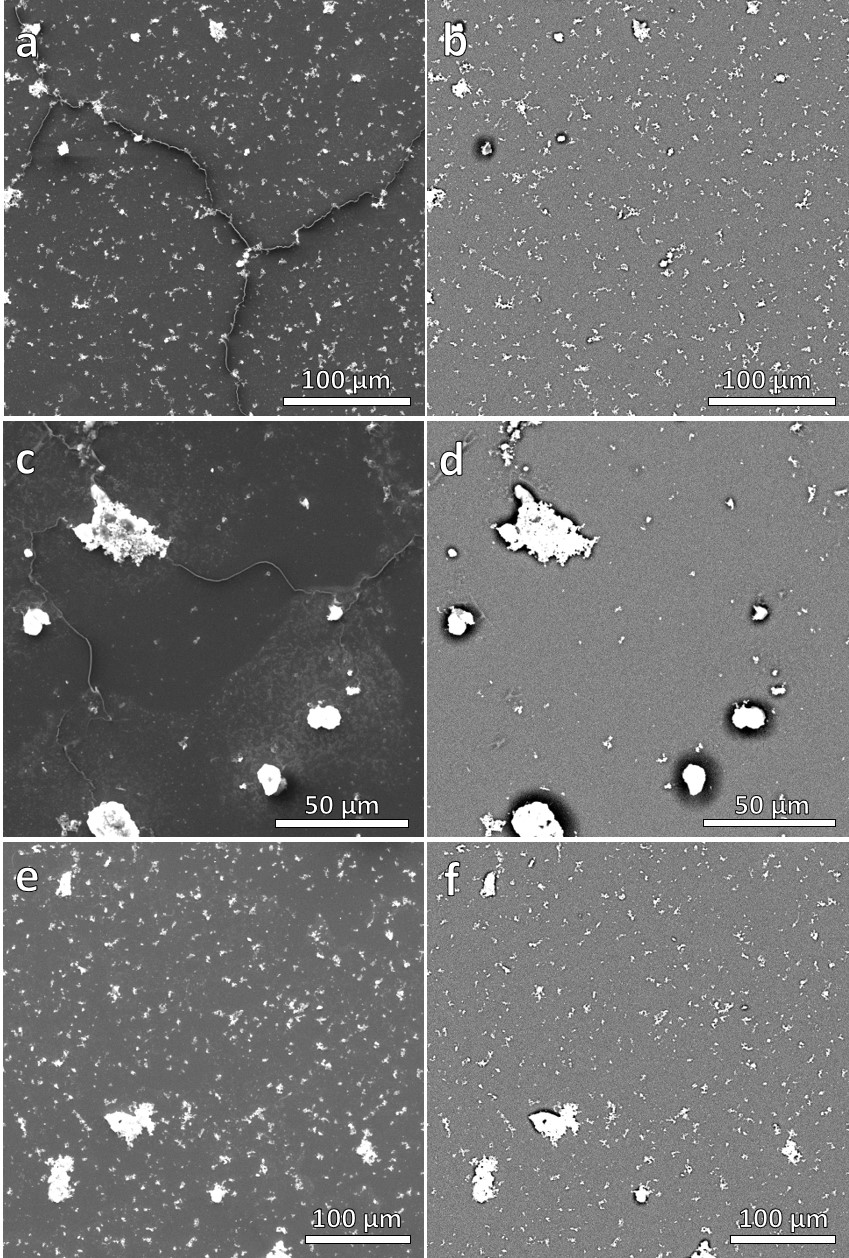


**Figure S3.** SEM micrographs for samples PNA_1 (a-b), PNA_2 (c-d) and PNA_3 (e-f). Topographical images in a, c and e panels were recorded with secondary electrons, while Z-sensitive images in panels b, d and f were recorded with backscattered electrons.


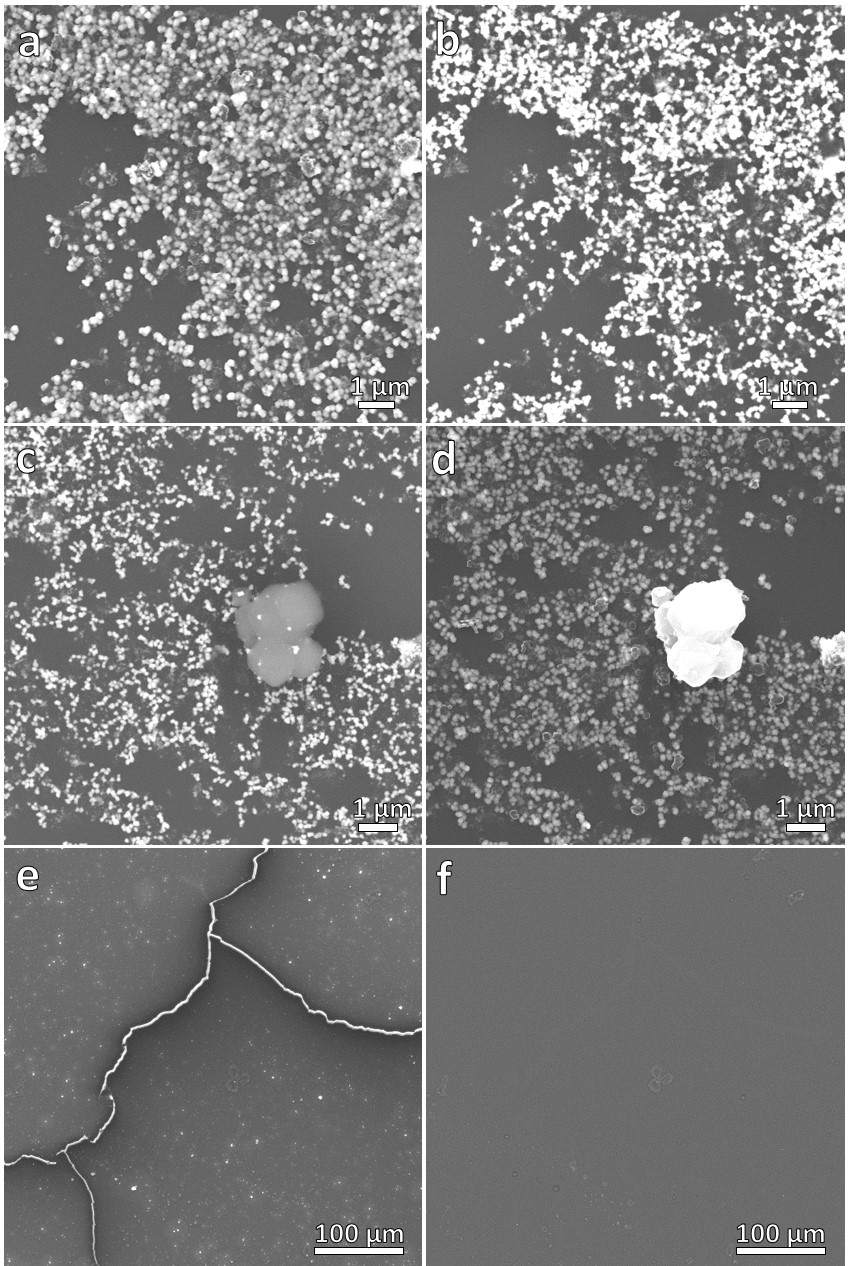


**Figure S4.** SEM micrographs for samples PNO_1 (a-b), PNO_2 (c-d) and PNO_3 (e-f). Topographical images in a, c and e panels were recorded with secondary electrons, while Z-sensitive images in panels b, d and f were recorded with backscattered electrons.

**Figure S5.** DLS measurements performed at room temperature for samples PNO_2 (above) and PNA_2 (below).


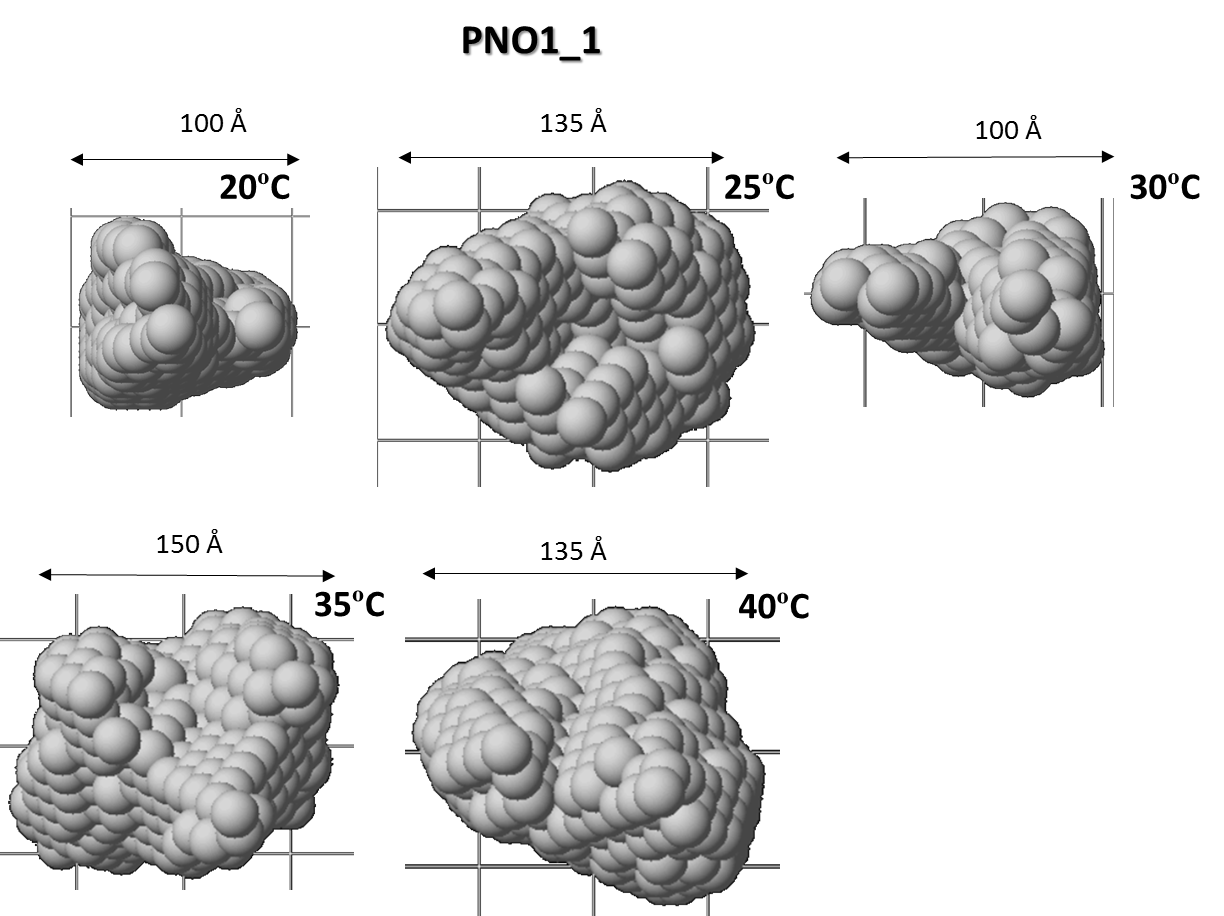


**Figure S6.** DAMMIN low-resolution structures reconstructed from SAXS patterns for PNO1_1 at different temperatures. Grid: 50 Å.


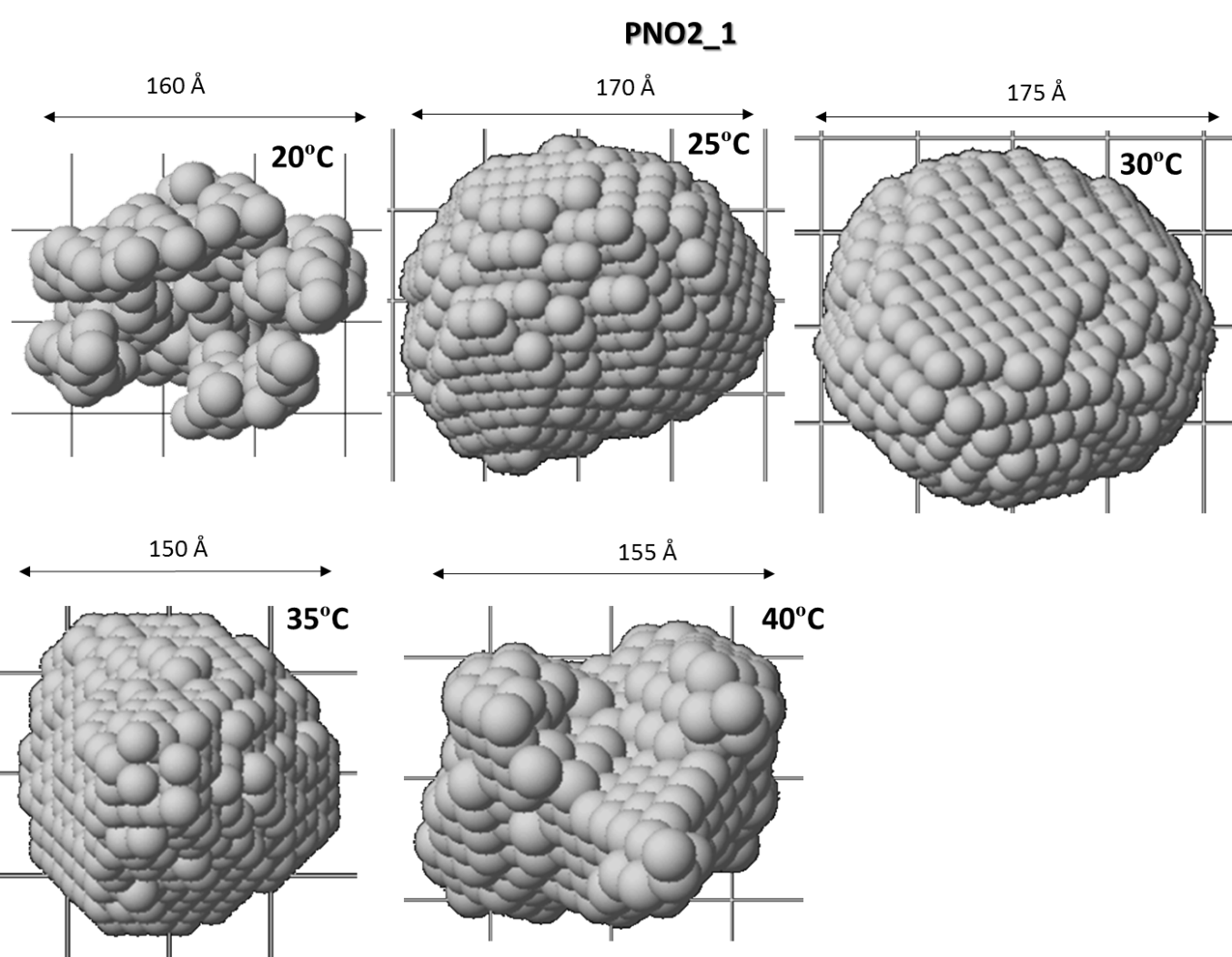


**Figure S7.** DAMMIN low-resolution structures reconstructed from SAXS patterns for PNO2_1 at different temperatures. Grid: 50 Å.


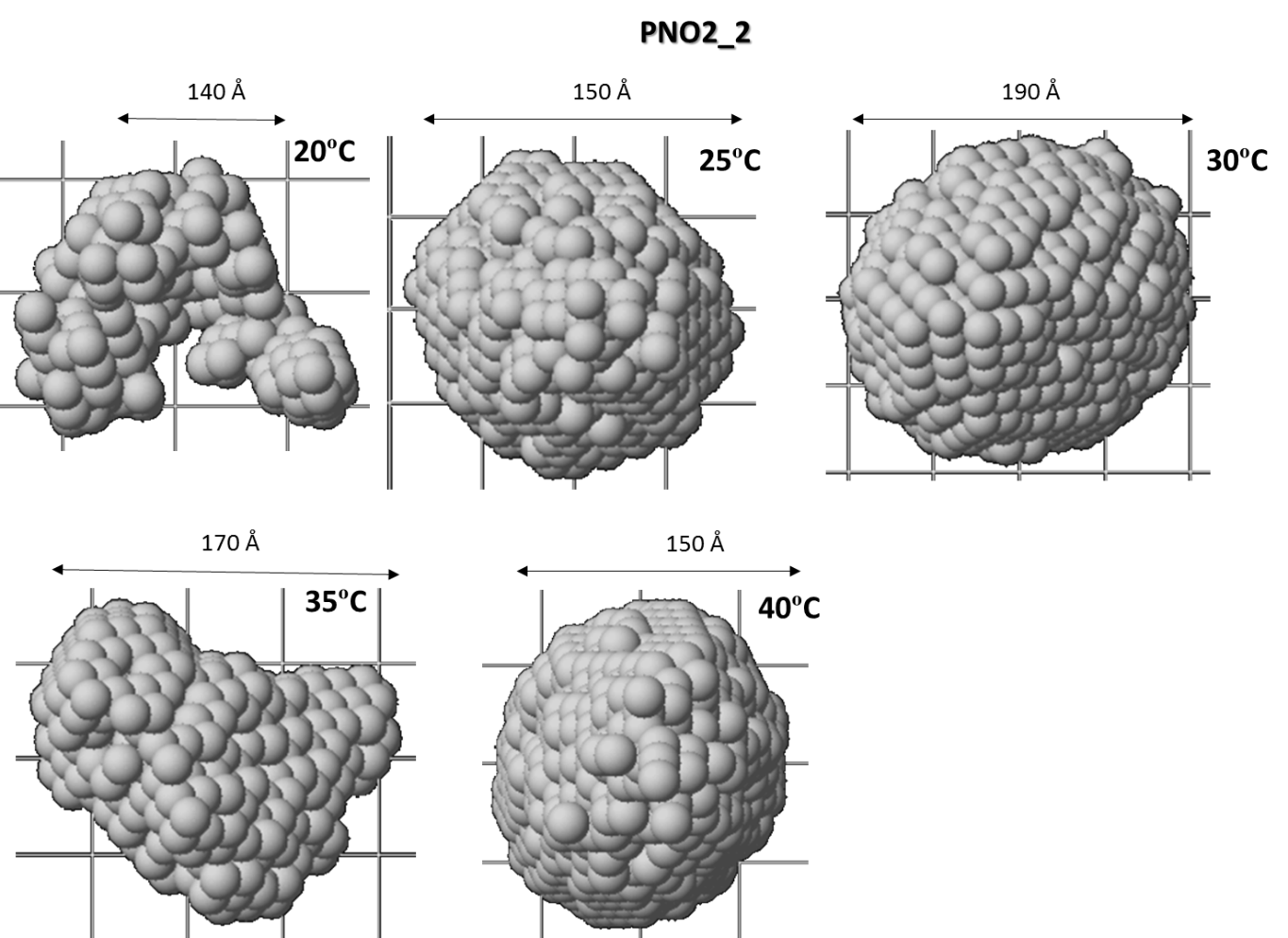


**Figure S8.** DAMMIN low-resolution structures reconstructed from SAXS patterns for PNO2_2 at different temperatures. Grid: 50 Å.


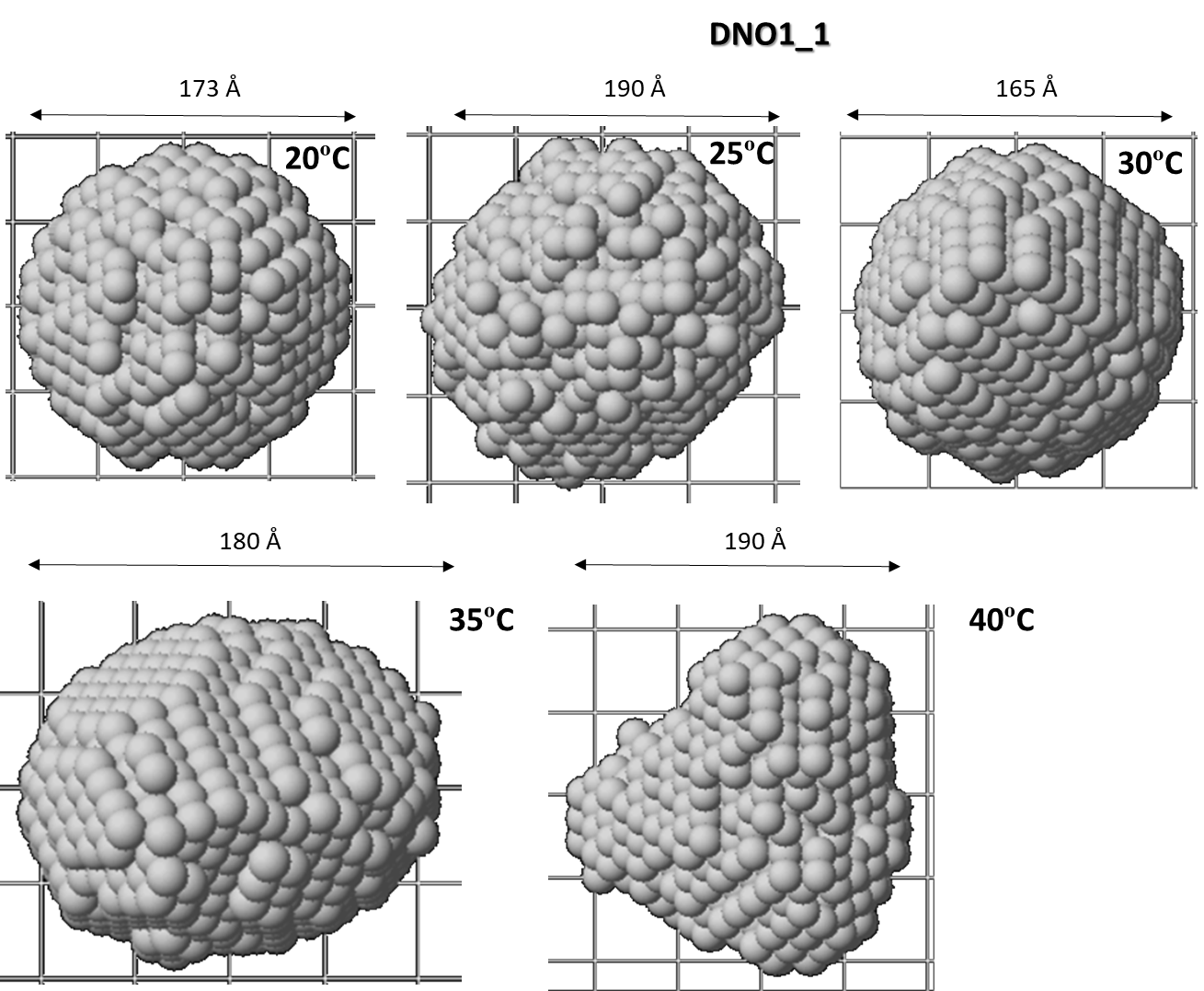


**Figure S9.** DAMMIN low-resolution structures reconstructed from SAXS patterns for DNO1_1 at different temperatures. Grid: 50 Å.


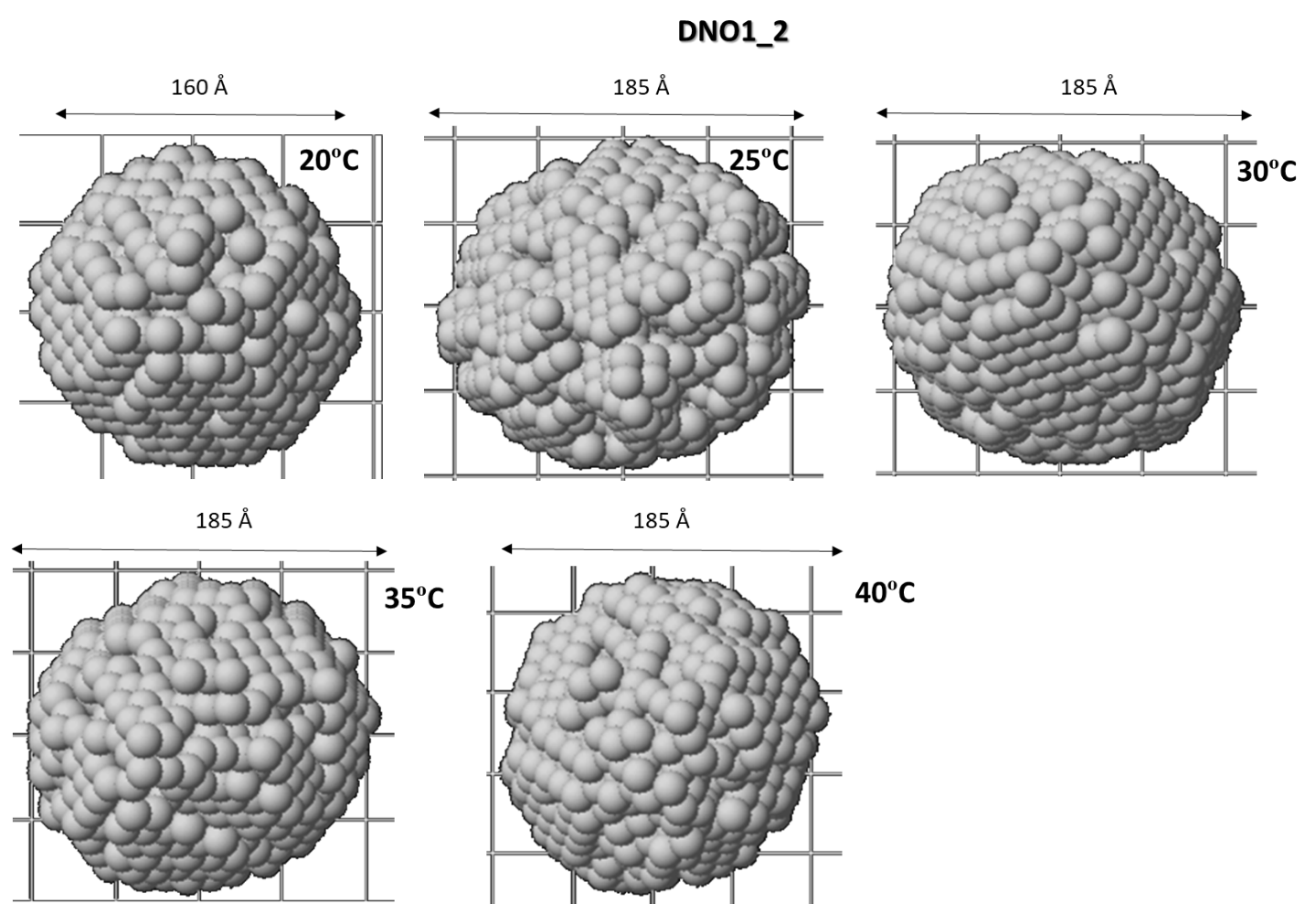


**Figure S10.** DAMMIN low-resolution structures reconstructed from SAXS patterns for DNO1_2 at different temperatures. Grid: 50 Å.


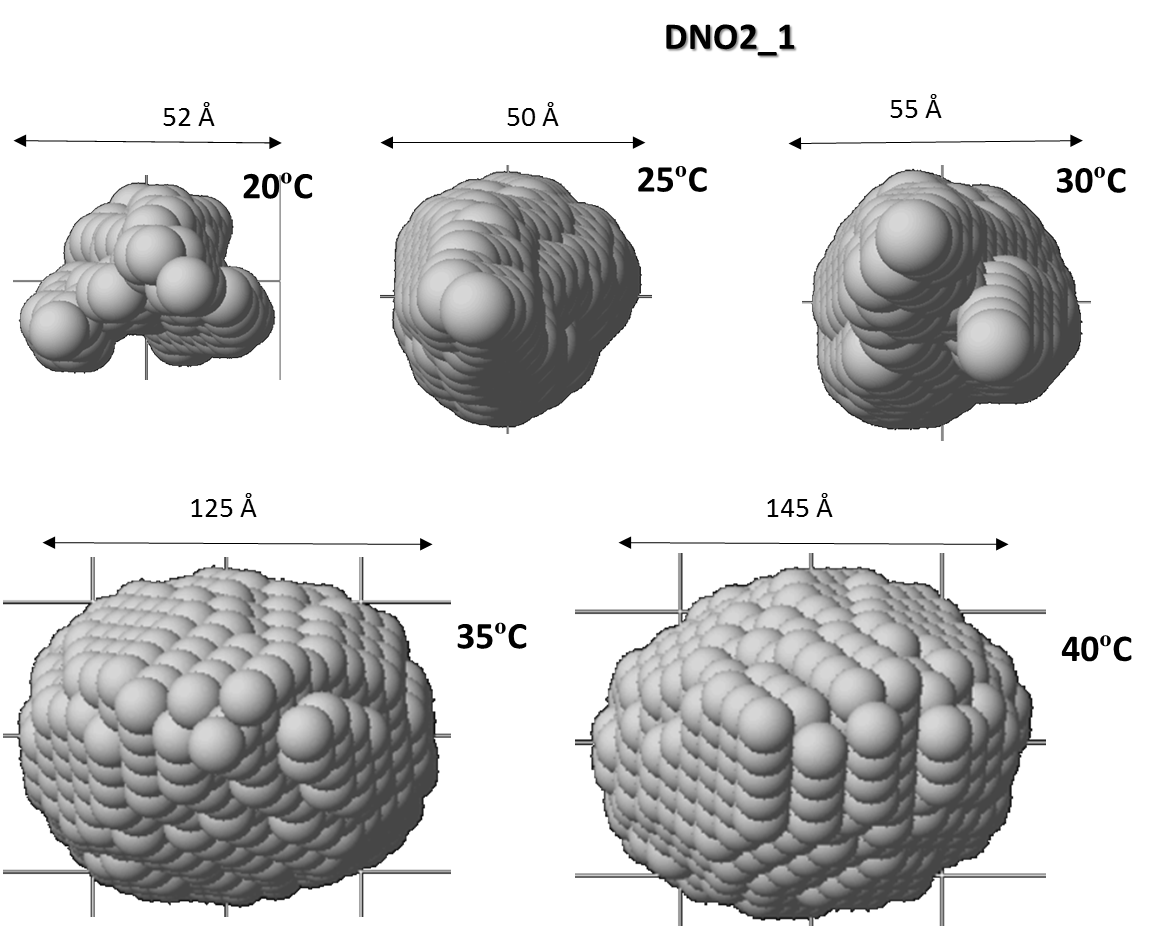


**Figure S11.** DAMMIN low-resolution structures reconstructed from SAXS patterns for DNO2_1 at different temperatures. Grid: 50 Å.


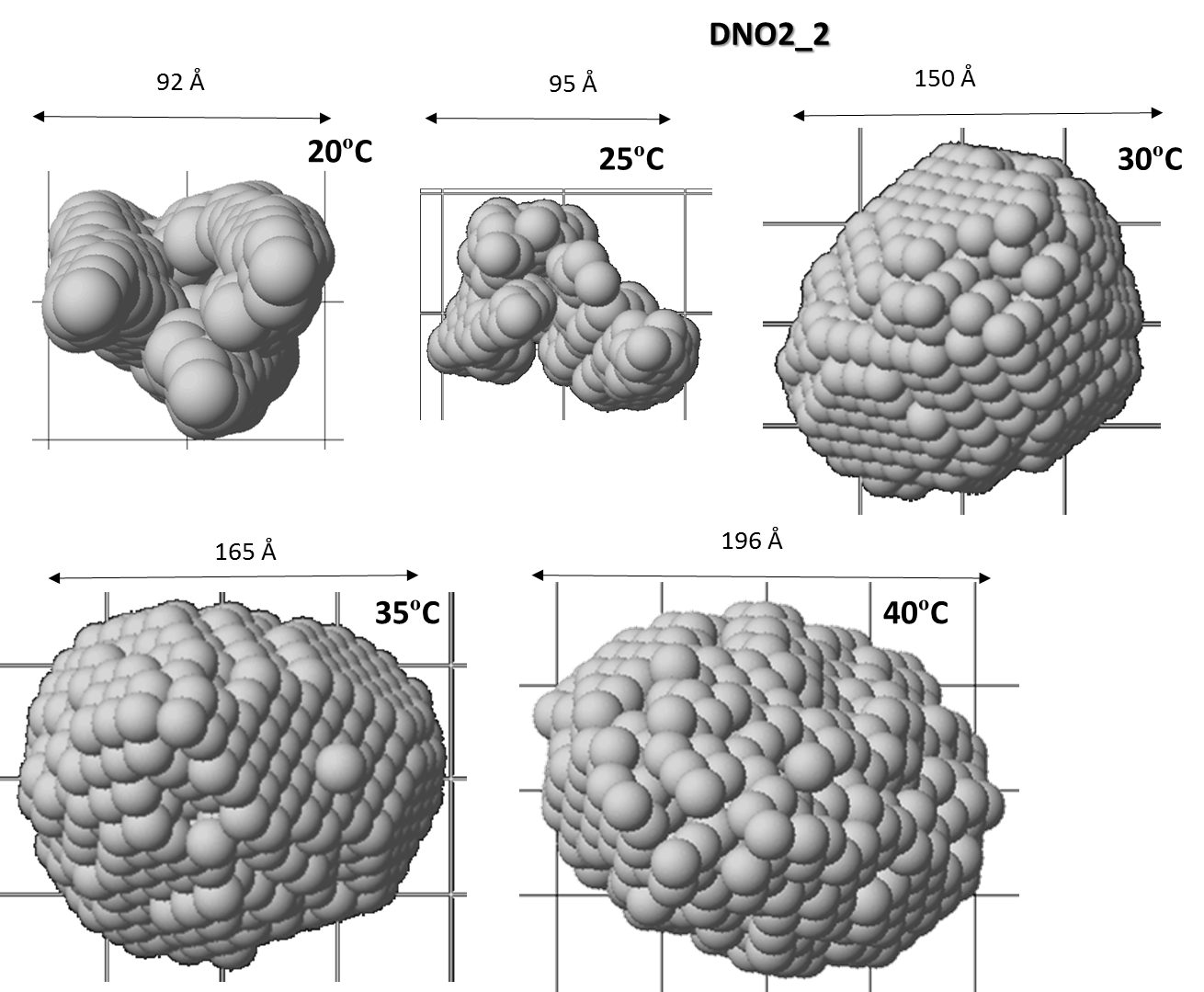


**Figure S12.** DAMMIN low-resolution structures reconstructed from SAXS patterns for DNO2_2 at different temperatures. Grid: 50 Å.


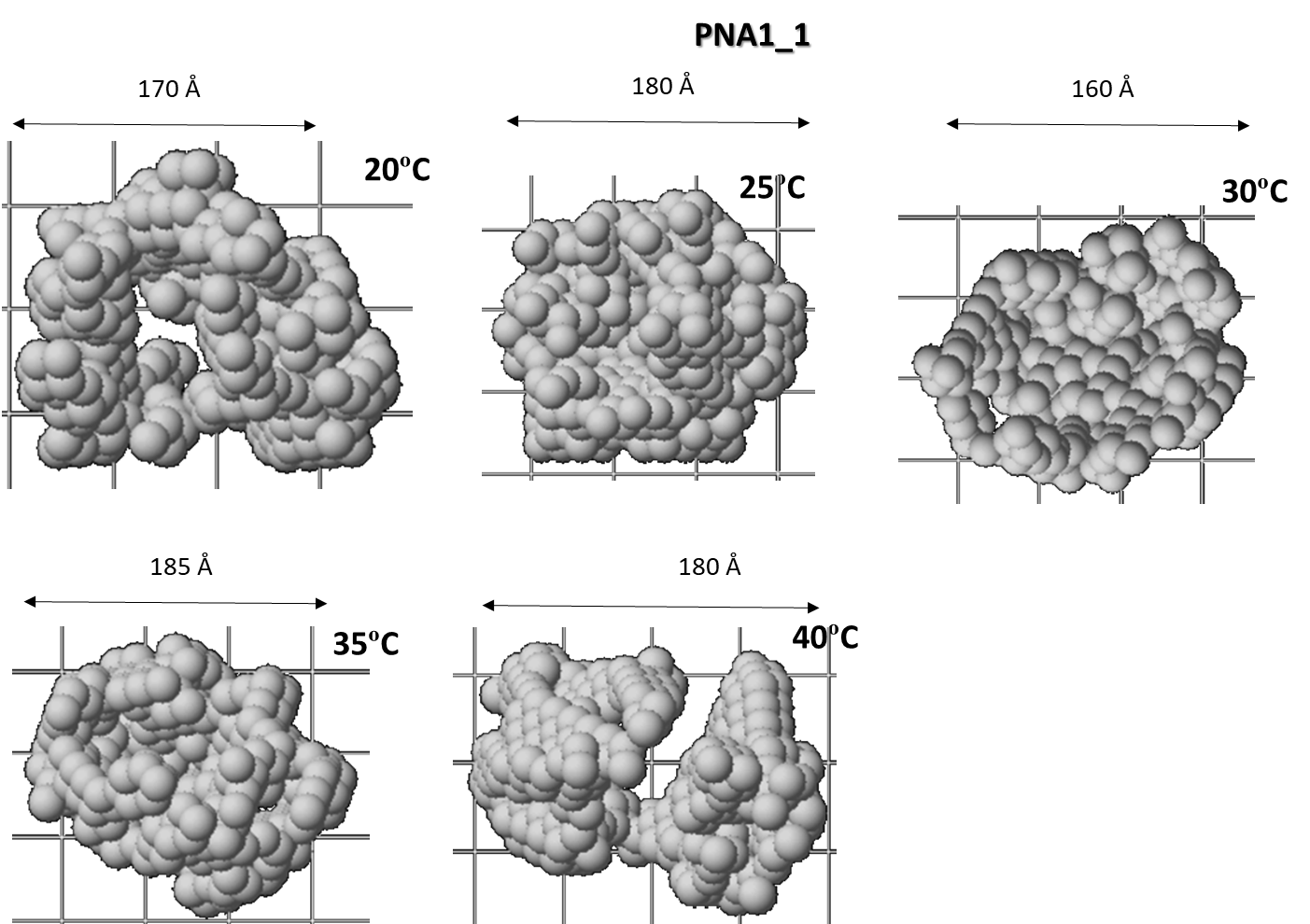


**Figure S13.** DAMMIN low-resolution structures reconstructed from SAXS patterns for PNA1_1 at different temperatures. Grid: 50 Å.


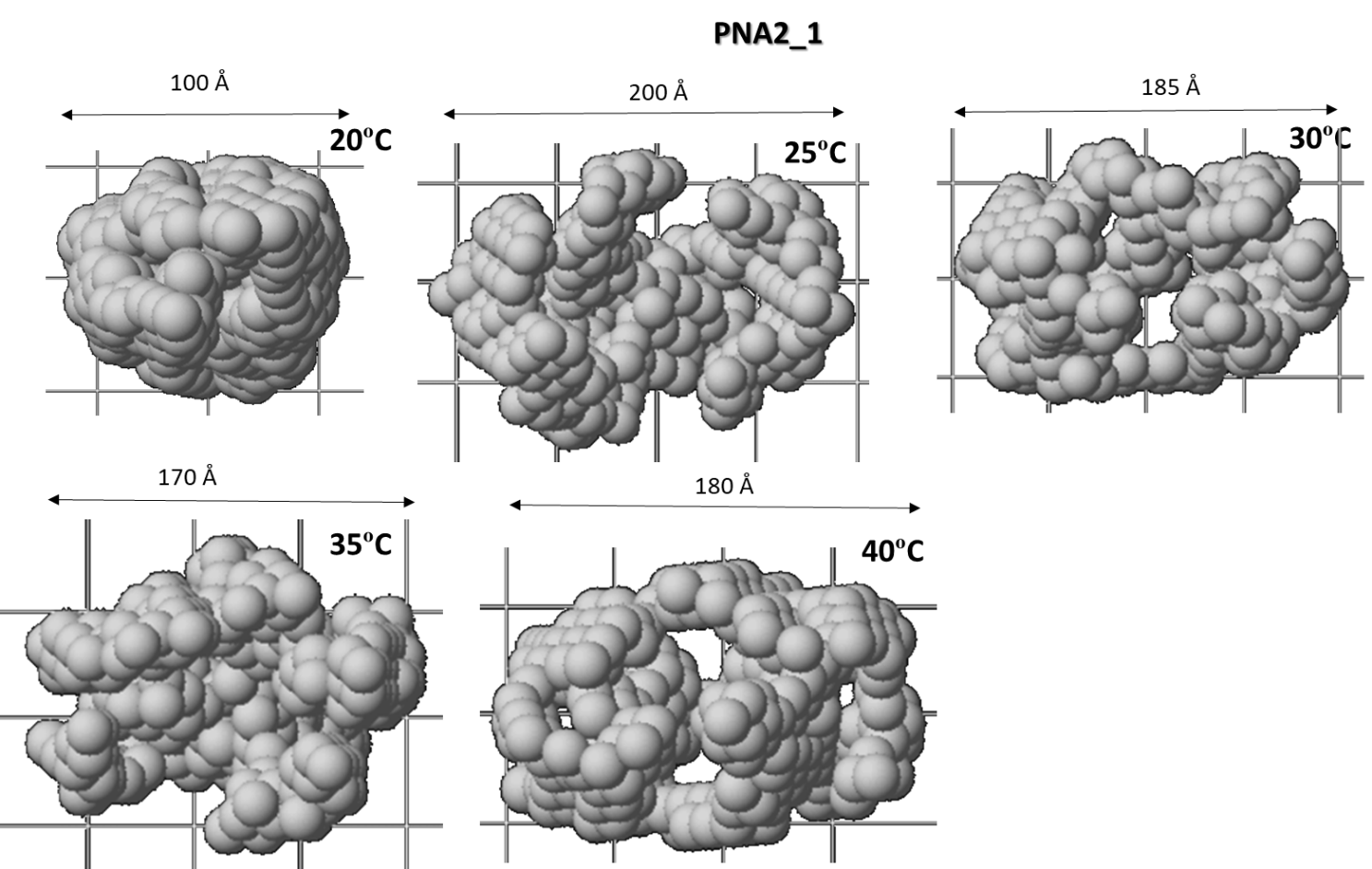


**Figure S14.** DAMMIN low-resolution structures reconstructed from SAXS patterns for PNA2_1 at different temperatures. Grid: 50 Å.


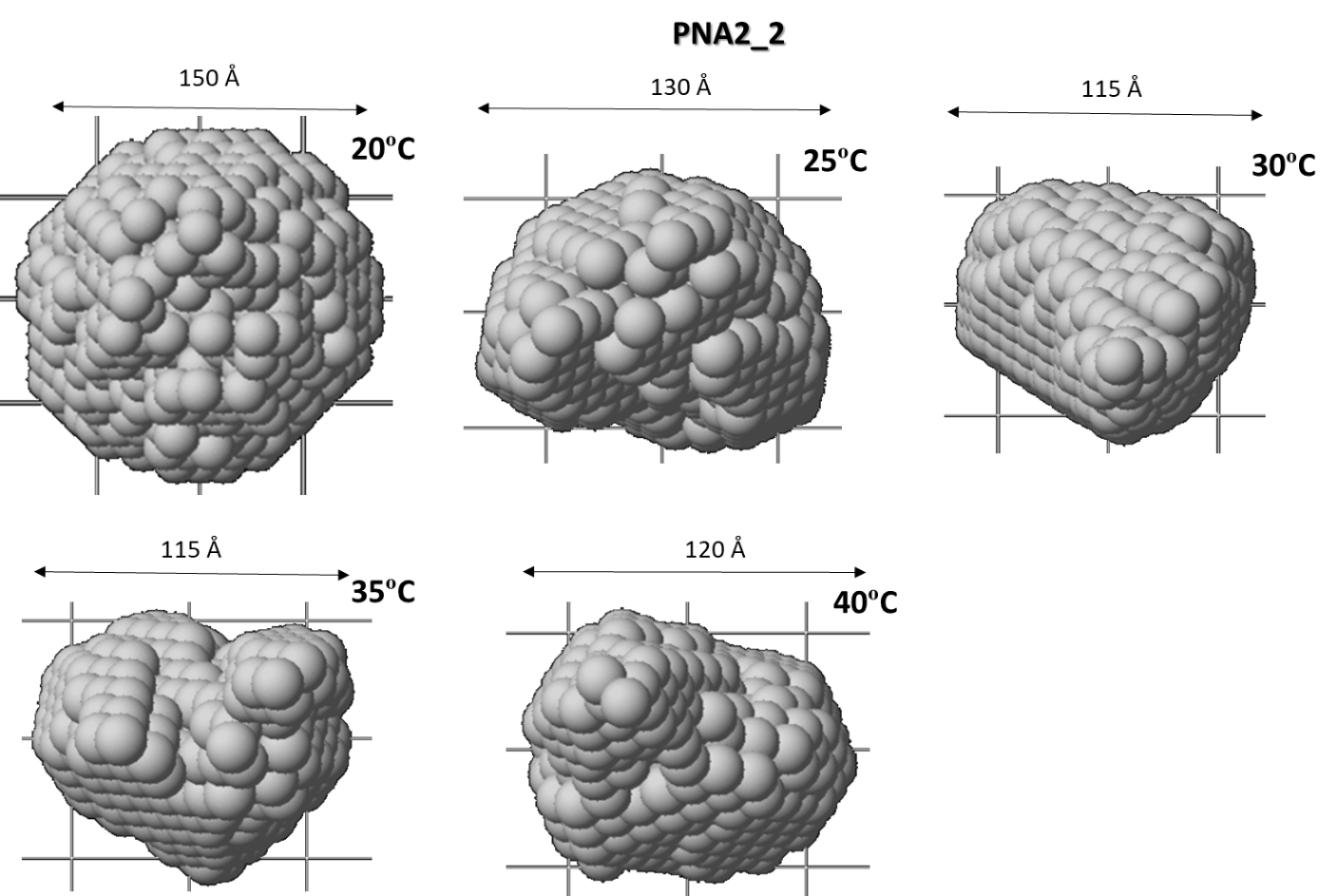


**Figure S15.** DAMMIN low-resolution structures reconstructed from SAXS patterns for PNA2_2 at different temperatures. Grid: 50 Å.


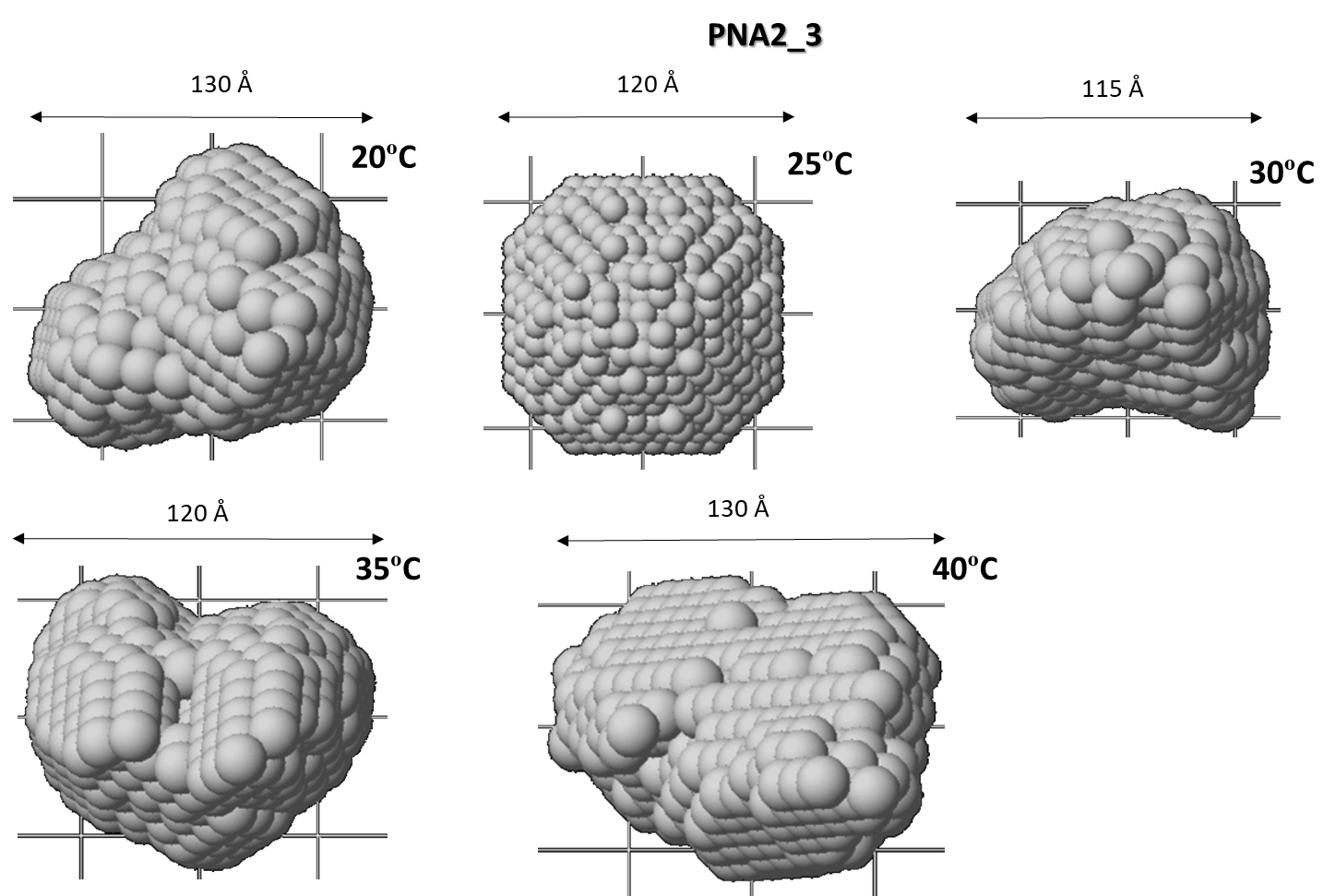


**Figure S16.** DAMMIN low-resolution structures reconstructed from SAXS patterns for PNA2_3 at different temperatures. Grid: 50 Å.


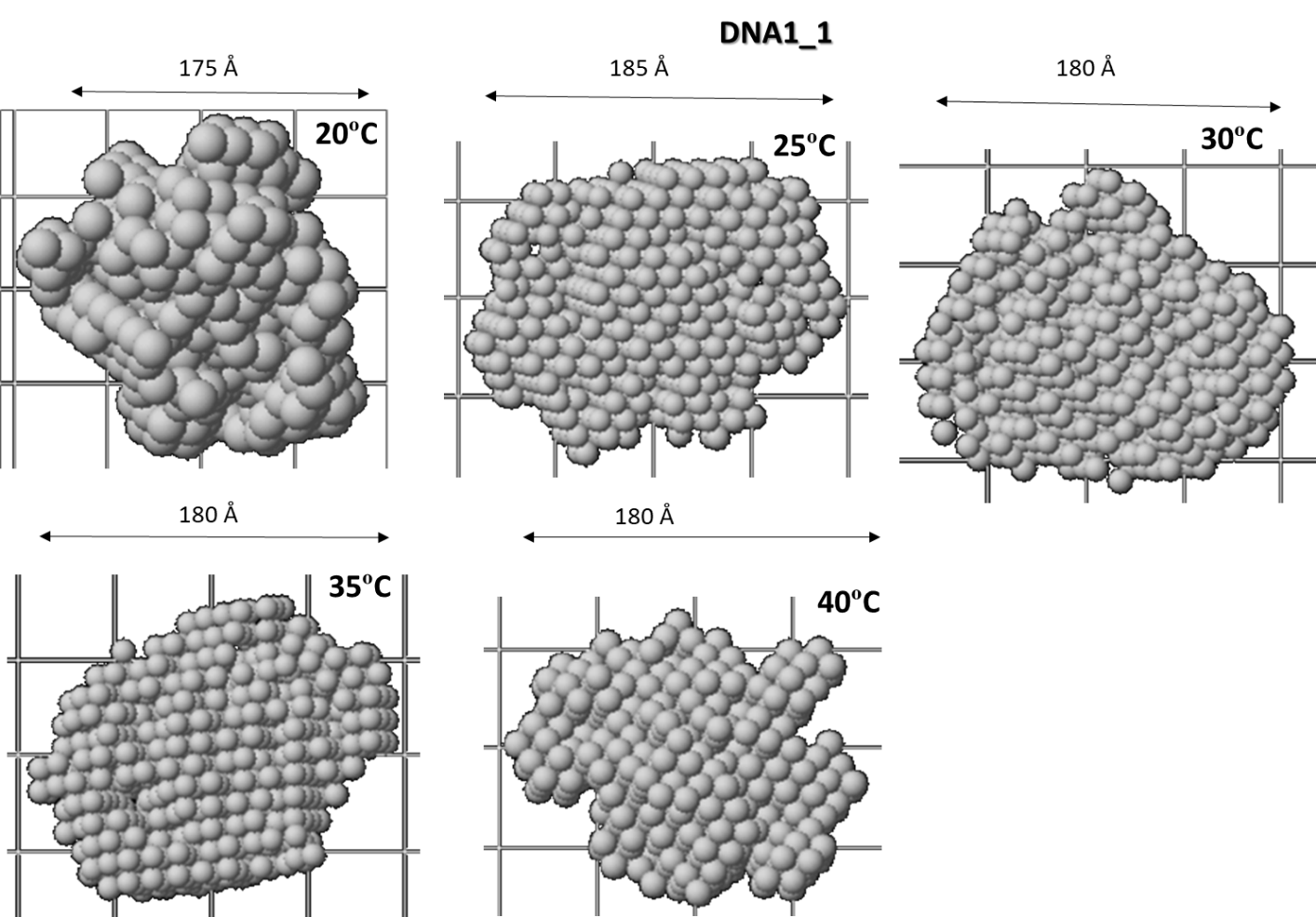


**Figure S17.** DAMMIN low-resolution structures reconstructed from SAXS patterns for DNA1_1 at different temperatures. Grid: 50 Å.


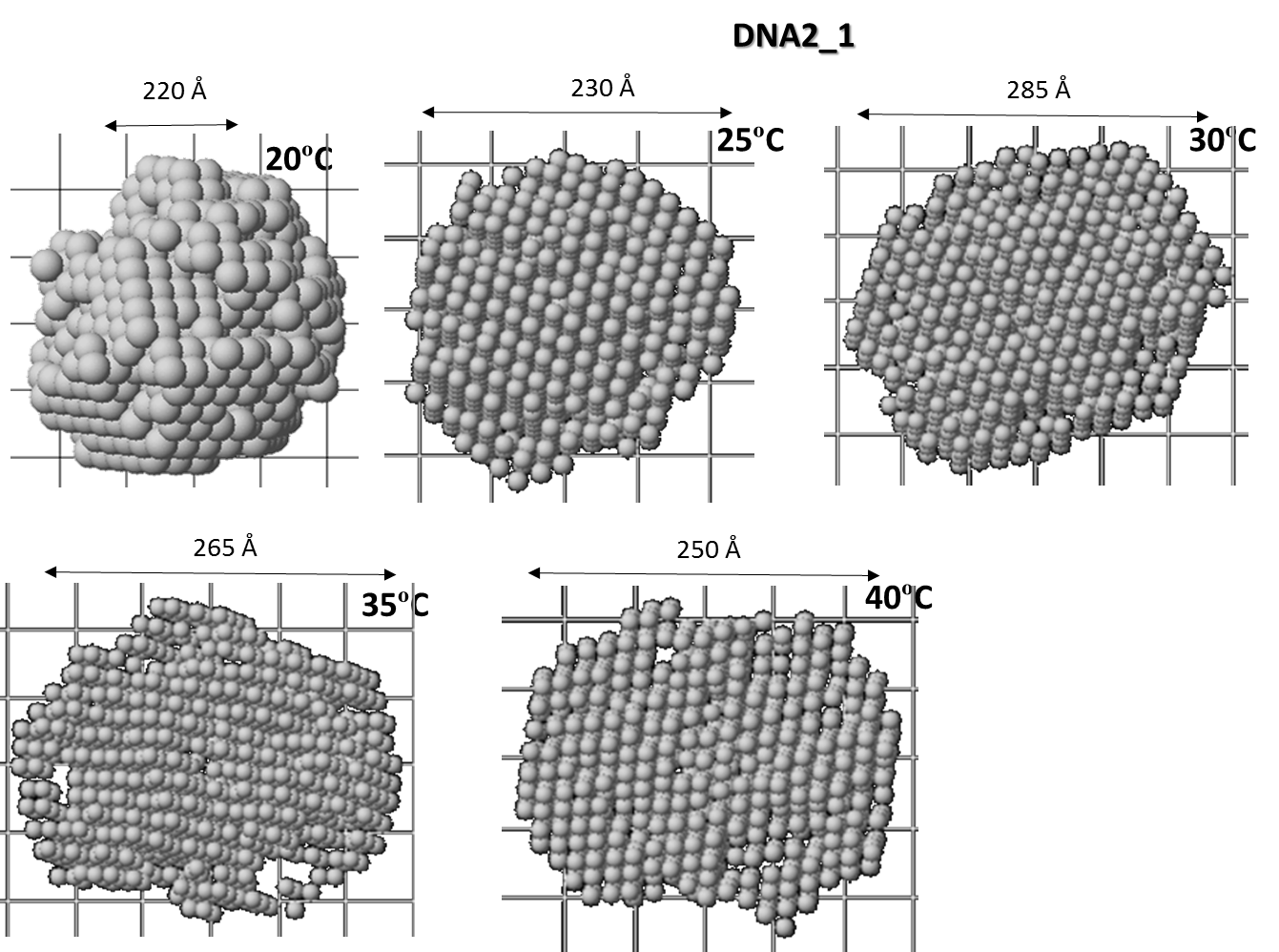


**Figure S18.** DAMMIN low-resolution structures reconstructed from SAXS patterns for DNA2_1 at different temperatures. Grid: 50 Å.
